# Transformer-Based Multi-Task Learning with a Task-Aware Weighted Ensemble for Simultaneous Age Estimation and Gender Classification from Facial Images

**DOI:** 10.3390/s26185982

**Published:** 2026-09-21

**Authors:** Sümeyye Sarıateş, Erdal Özbay

**Affiliations:** Department of Computer Engineering, Firat University, 23119 Elazig, Türkiye; sumeyyesariates@gmail.com

**Keywords:** age estimation, gender classification, facial demographic analysis, multi-task learning, vision transformer, weighted ensemble

## Abstract

**Highlights:**

**What are the main findings?**

- A multi-task transformer framework was developed for simultaneous age estimation and gender classification from facial images.
- The proposed weighted ensemble achieved the best point estimates among the evaluated ensemble strategies, with 4.8905 MAE and 6.4674 RMSE for age estimation, and 94.86% accuracy and 95.17% F1-score for gender classification.

**What are the implications of the main findings?**

- Task-aware weighted ensemble learning can effectively combine the strengths of different transformer architectures for facial demographic analysis.
- The proposed framework provides a flexible approach for multi-task facial demographic analysis.

**Abstract:**

Accurate age estimation and gender classification from facial images remain challenging tasks owing to substantial variations in facial appearance caused by pose, illumination, expression, occlusion, image quality, and age-related morphological changes. Although transformer-based architectures have recently demonstrated remarkable performance in facial analysis, effectively exploiting their complementary representation capabilities within a unified multi-task framework remains an open research problem. To address this limitation, this study proposes a transformer-based Multi-task Learning (MTL) framework that jointly performs age estimation and gender classification using a shared feature extraction backbone and task-specific prediction heads. Three state-of-the-art vision transformers, namely Vision Transformer (ViT), Shifted Window Transformer (Swin Transformer), and Data-efficient Image Transformer (DeiT), were independently implemented within the proposed MTL architecture to learn both global contextual information and local facial characteristics. Furthermore, two ensemble learning strategies, namely standard ensemble and Task-aware Weighted Ensemble (TAWE), were developed to exploit the complementary strengths of the individual transformer models. Experiments were conducted on the publicly available UTKFace dataset comprising 15,503 facial images, which were divided into 10,385 training, 1833 validation, and 3285 test samples, while gender balancing was applied during model training to alleviate class imbalance. Age estimation performance was evaluated using Mean Absolute Error (MAE) and Root Mean Squared Error (RMSE), whereas gender classification performance was assessed using Accuracy and F1-score. Experimental results demonstrate that the proposed TAWE achieved the best point estimates among the evaluated ensemble strategies, obtaining an MAE of 4.8905, an RMSE of 6.4674, a gender classification accuracy of 94.86%, and an F1-score of 95.17% for the dataset-defined positive class. Compared with equal-weight averaging, TAWE provided a statistically significant improvement in gender classification accuracy, while the improvement in age estimation was modest and not statistically significant.

## 1. Introduction

Automatic facial analysis has become one of the most active research areas in computer vision owing to its wide range of applications in biometric authentication, intelligent surveillance, human–computer interaction, healthcare, digital forensics, demographic analytics, and personalized recommendation systems. Among various facial analysis tasks, age estimation and gender classification have attracted considerable attention because they provide valuable demographic information that can enhance decision-making processes in numerous real-world applications. Reliable estimation of these attributes enables adaptive human–machine interaction, improves identity verification systems, supports intelligent retail analytics, facilitates public security applications, and contributes to personalized digital services. Consequently, developing robust computational models capable of accurately estimating age and gender from unconstrained facial images has become an important research topic in both academia and industry [1].

Despite significant advances in deep learning, automatic age estimation and gender classification remain challenging problems. Facial appearance is influenced by numerous intrinsic and extrinsic factors, including pose variations, illumination changes, facial expressions, occlusions, ethnicity, cosmetics, facial hair, accessories, image resolution, and sensor quality. Furthermore, aging is a continuous biological process characterized by highly nonlinear and person-specific morphological changes, making age estimation considerably more difficult than conventional image classification problems. Individuals with similar chronological ages may exhibit substantially different facial characteristics due to genetic, environmental, and lifestyle factors. Conversely, people from different age groups may share similar facial appearances, increasing the complexity of age prediction models. Gender classification, although generally formulated as a binary classification problem, is likewise affected by substantial intra-class variability and inter-class similarity, particularly in unconstrained environments. These challenges necessitate the development of learning frameworks capable of extracting highly discriminative yet generalized facial representations under diverse imaging conditions [1].

Early deep learning studies primarily relied on Convolutional Neural Networks (CNNs), which demonstrated remarkable improvements over traditional handcrafted feature extraction techniques. CNN-based models automatically learn hierarchical visual representations from raw facial images and have significantly advanced the state of the art in facial demographic analysis. Their ability to capture low-level texture information together with increasingly abstract semantic representations has established deep learning as the dominant paradigm for age estimation and gender classification. Nevertheless, conventional CNN architectures mainly focus on local receptive fields, which may limit their capability to model long-range spatial dependencies among different facial regions. As a result, representing complex global facial structures that jointly characterize demographic attributes remains a challenging problem, motivating the investigation of alternative architectures with stronger contextual modeling capabilities [1].

To improve feature learning efficiency, researchers have increasingly explored Multi-Task Learning (MTL) frameworks that jointly optimize multiple related facial analysis tasks within a unified architecture. Instead of learning each task independently, MTL enables the model to exploit shared visual representations while simultaneously preserving task-specific characteristics. Since age estimation and gender classification originate from the same facial image and share numerous discriminative visual cues, jointly learning these tasks can reduce redundant feature extraction, improve generalization capability, and enhance computational efficiency. Consequently, MTL has emerged as an attractive solution for facial demographic analysis, particularly when different tasks exhibit strong semantic correlations.

One of the pioneering transformer-based MTL studies in facial analysis was introduced by Qin et al., who proposed SwinFace, a unified framework that simultaneously performs multiple facial analysis tasks, including face recognition, facial expression recognition, age estimation, and facial attribute prediction. Their architecture employs a shared Shifted Window Transformer (Swin Transformer) backbone together with task-specific prediction branches, enabling efficient knowledge sharing across heterogeneous facial analysis tasks. Experimental findings demonstrated that shared representation learning substantially improves feature discriminability while reducing computational redundancy, highlighting the effectiveness of transformer-based MTL for complex facial analysis problems [2].

Similarly, Liao et al. investigated joint age and gender prediction using an MTL framework combined with multi-instance feature fusion. Their study demonstrated that learning age and gender simultaneously allows the model to exploit complementary demographic information, thereby improving overall prediction performance. By integrating shared feature representations with task-specific learning modules, the proposed framework achieved more robust demographic attribute estimation than independently trained models. These findings further emphasize that age estimation and gender classification should not be regarded as completely independent problems but rather as mutually beneficial learning tasks that can effectively share common facial representations [3].

The effectiveness of shared representation learning was further demonstrated by Han et al., who introduced a deep MTL framework for heterogeneous facial attribute estimation. Their architecture simultaneously estimated multiple demographic attributes, including age, gender, race, and additional facial characteristics, within a unified deep neural network. While lower network layers extracted common facial representations, higher layers were specialized for individual prediction tasks, enabling efficient knowledge transfer among heterogeneous facial attributes. This study confirmed that jointly optimizing multiple facial analysis tasks leads to stronger feature representations and improved generalization compared with conventional single-task learning approaches [4].

Another influential study in this area is HyperFace, proposed by Ranjan et al., which integrated face detection, facial landmark localization, head pose estimation, and gender recognition into a single deep learning architecture. Unlike conventional pipelines that independently solved each facial analysis task, HyperFace exploited intermediate feature representations to simultaneously capture both local facial details and global structural information. The experimental results demonstrated that shared multi-task optimization substantially improved overall performance, illustrating the advantages of learning correlated facial analysis tasks within a unified framework [5].

While MTL focuses on improving shared representation learning, significant efforts have also been devoted to developing more effective age estimation algorithms. Among the earliest and most influential deep learning approaches, DEX introduced a novel formulation by transforming age estimation into a classification problem and subsequently converting classification probabilities into continuous age predictions using the expected value. Built upon the VGG-16 architecture, DEX significantly improved age estimation accuracy and established deep learning as the dominant methodology for apparent age prediction from facial images [6].

Following this direction, SSR-Net introduced a lightweight yet highly efficient CNN architecture specifically designed for age estimation. Instead of employing conventional regression strategies, SSR-Net utilized a soft stagewise regression mechanism that progressively refined age predictions through hierarchical decision stages. Despite its compact architecture and relatively small computational complexity, SSR-Net achieved competitive estimation performance, demonstrating that carefully designed network architectures can successfully balance prediction accuracy and computational efficiency. This work also highlighted the importance of architectural optimization in practical age estimation systems, particularly for real-time applications with limited computational resources [7].

Although CNN-based models substantially advanced facial demographic analysis, their inherent locality restricts the ability to model long-range dependencies across facial regions. Recently, the remarkable success of Transformer architectures in computer vision has opened new opportunities for addressing this limitation. By employing self-attention mechanisms, Vision Transformers (ViT) establish global interactions among image patches and learn richer contextual representations than conventional convolutional networks. Consequently, transformer-based models have rapidly gained attention for various facial analysis tasks, including age estimation, gender classification, facial recognition, expression analysis, and facial attribute prediction [8].

Among transformer architectures, the ViT represented a major breakthrough by reformulating image analysis as a sequence modeling problem. Instead of processing local convolutional kernels, ViT divides an image into fixed-size patches and applies the self-attention mechanism to capture long-range dependencies among all image regions simultaneously. This global modeling capability enables the network to learn holistic facial representations that may be particularly beneficial for demographic analysis, where discriminative information is often distributed across multiple facial regions rather than concentrated in localized textures [9].

The limitations of conventional ViTs in modeling local image structures motivated the development of the Swin Transformer. Unlike ViT, Swin Transformer introduces hierarchical feature extraction through shifted local attention windows, enabling efficient modeling of both local textures and global contextual relationships. Such hierarchical representation learning is particularly advantageous for facial analysis because age-related characteristics—including wrinkles, skin texture, facial contours, and localized morphological changes—often appear at multiple spatial scales. Consequently, Swin Transformer has demonstrated remarkable effectiveness across numerous vision tasks requiring fine-grained structural representation [10].

In addition to architectural innovations, efficient transformer training has also become an important research direction. The Data-efficient Image Transformer (DeiT) addressed this issue by introducing a knowledge distillation strategy that substantially reduces the amount of training data required by transformer models while preserving competitive performance. Through teacher–student learning and optimized training procedures, DeiT demonstrated that ViTs can achieve high accuracy even under relatively limited data conditions. This property is particularly attractive for facial demographic analysis, where collecting large-scale, well-balanced datasets covering all age groups and demographic variations remains a challenging task [11].

Recent comprehensive surveys indicate that facial demographic analysis has evolved from single-attribute prediction toward integrated multi-attribute learning frameworks capable of simultaneously estimating multiple facial characteristics. Gupta and Nain comprehensively reviewed both single-task and multi-task age and gender prediction methods and concluded that deep learning models significantly outperform conventional handcrafted feature extraction techniques owing to their superior representation learning capability. More importantly, their review emphasized that jointly modeling correlated demographic attributes, such as age and gender, enables the extraction of richer facial representations and improves the generalization capability of facial analysis systems. This observation further supports the growing tendency toward unified learning frameworks that simultaneously exploit shared facial characteristics while preserving task-specific discriminative information [12].

The theoretical foundation of this paradigm is provided by MTL, which enables multiple related tasks to be optimized simultaneously through a shared feature representation space. Rather than independently learning separate feature extractors for each prediction task, MTL facilitates knowledge transfer among correlated tasks, reduces the risk of overfitting, improves data efficiency, and enhances model generalization. Zhang and Yang demonstrated that jointly learning related tasks enables neural networks to exploit complementary information that may not be sufficiently captured in single-task optimization. Since age estimation and gender classification originate from the same facial appearance and share numerous visual characteristics, including facial geometry, texture, structural proportions, and aging-related morphological patterns, they naturally constitute highly correlated learning objectives that are particularly well suited for multi-task optimization [13].

Although considerable progress has been achieved in recent years, several important limitations remain in the existing literature. First, many studies focus exclusively on either age estimation or gender classification, thereby failing to exploit the complementary information shared between these two demographic attributes. Second, even among MTL approaches, most existing methods employ a single backbone architecture, assuming that one feature extraction model is equally suitable for all prediction tasks. However, different transformer architectures possess distinct representation characteristics. For example, architectures emphasizing global contextual relationships may be more effective for capturing facial geometry, whereas hierarchical attention mechanisms may better represent localized aging cues such as wrinkles, skin texture, and facial contours. Consequently, relying on a single backbone may restrict the overall representation capability of multi-task facial analysis systems.

Although recent studies have demonstrated the effectiveness of transformer architectures and MTL for facial analysis, their methodological focus varies considerably. Some approaches employ transformer-based backbones within a single multi-task framework, whereas others rely on conventional convolutional architectures or address only one demographic prediction task. Furthermore, most existing studies utilize a single feature extraction backbone and adopt either independent prediction heads or conventional fusion strategies, limiting their ability to exploit complementary representation characteristics across different transformer architectures. A critical comparison of representative studies is presented in Table 1.

As summarized in Table 1, the representative studies considered in this comparison do not combine multiple transformer backbones within a unified MTL framework together with task-specific validation-based weighting for simultaneous continuous age estimation and gender classification. Existing transformer-based approaches typically rely on a single backbone architecture, implicitly assuming that one representation model is equally suitable for all demographic prediction tasks. However, the experimental findings of the present study demonstrate that different transformer architectures exhibit complementary strengths. This observation motivated the development of the proposed Task-Aware Weighted Ensemble (TAWE), which exploits these complementary characteristics through task-specific validation-based weighting.

Beyond these architectural limitations, an important research gap still exists regarding how heterogeneous transformer representations should be effectively integrated within a unified multi-task facial analysis framework. Most existing studies implicitly assume that a single backbone architecture can simultaneously provide optimal feature representations for both age estimation and gender classification. However, these two tasks exhibit fundamentally different learning characteristics. Age estimation requires fine-grained modeling of continuous age-related facial changes, whereas gender classification primarily relies on capturing global semantic facial structures. Consequently, different transformer architectures may demonstrate complementary strengths for different prediction tasks rather than a universally superior performance. Despite this observation, relatively little attention has been devoted to task-aware ensemble mechanisms capable of exploiting these complementary transformer characteristics within a shared MTL framework.

Another limitation concerns the utilization of ensemble learning. Existing ensemble approaches generally combine multiple model predictions using equal-weight averaging or conventional voting strategies without considering the heterogeneous strengths of individual models for different prediction tasks. In practice, however, a transformer architecture that performs optimally for age estimation may not necessarily achieve the highest performance in gender classification. Therefore, assigning identical contributions to all models for all tasks may prevent the ensemble from fully exploiting the complementary learning characteristics of different transformer architectures. Despite the remarkable advances in transformer-based computer vision, task-aware ensemble learning for joint age estimation and gender classification remains insufficiently investigated.

**Figure 1:**
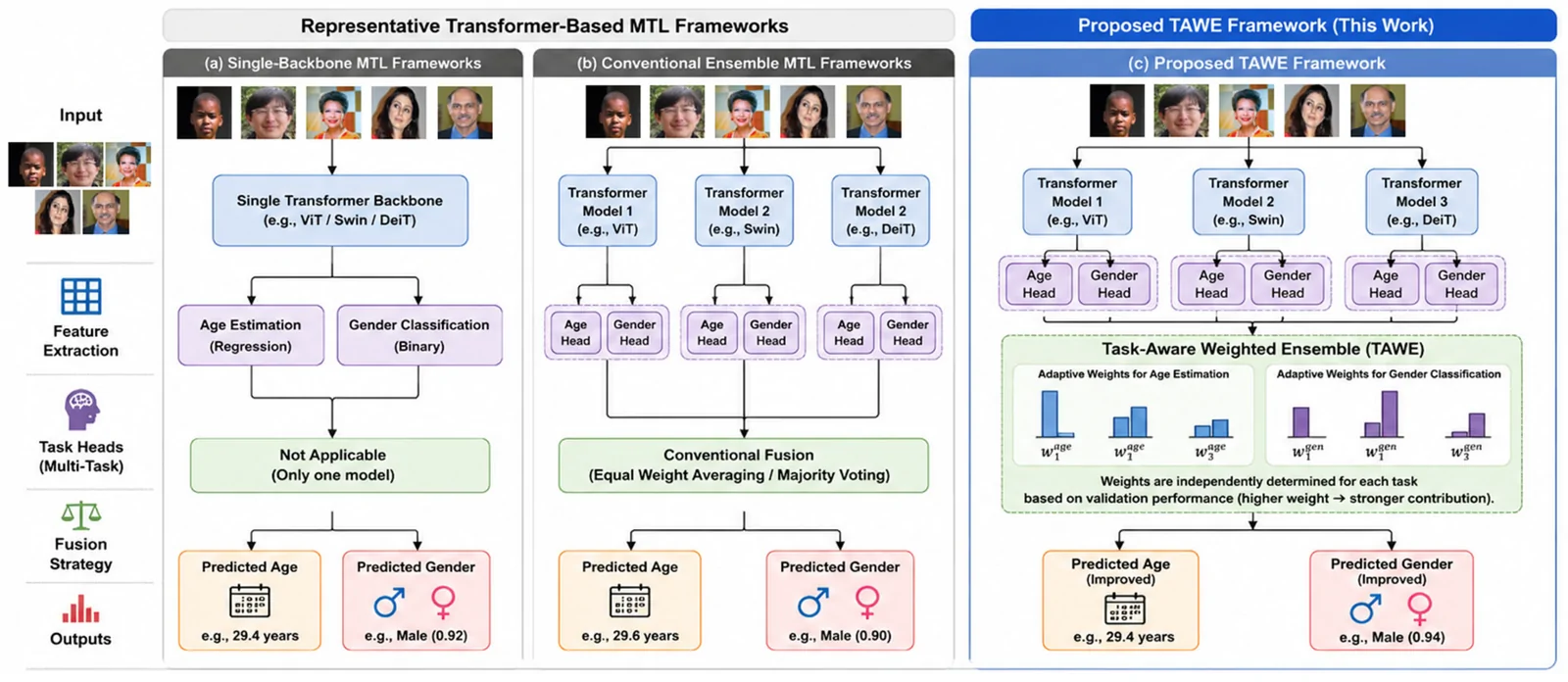
Comparison between representative transformer-based facial demographic analysis frameworks (**a**,**b**) and the proposed TAWE framework (**c**). Existing methods generally rely on a single transformer backbone or conventional ensemble learning, whereas the proposed framework combines complementary transformer architectures within a unified MTL architecture using task-specific validation-based weighting.

To better illustrate the research gap and highlight the methodological novelty of the proposed approach, Figure 1 presents a conceptual comparison between representative transformer-based multi-task facial analysis frameworks and the proposed TAWE framework. Unlike existing approaches that typically rely on a single transformer backbone or conventional equal-weight fusion strategies, the proposed framework explicitly exploits the complementary representation capabilities of multiple transformer architectures through task-specific validation-based weighting. This comparison provides the conceptual motivation underlying the proposed methodology and clarifies its distinction from representative transformer-based multi-task facial demographic analysis methods.

Motivated by these limitations, this study proposes a transformer-based MTL framework that extends existing facial demographic analysis methods in two important directions. First, instead of assuming that a single transformer backbone can optimally represent all demographic prediction tasks, three complementary transformer architectures (ViT, Swin Transformer, and DeiT) are systematically investigated within an identical MTL framework. Second, unlike conventional ensemble strategies that assign identical contributions to all models regardless of the target task, a task-aware validation-based ensemble strategy (TAWE) is introduced to assign independent model contributions according to validation performance. This design enables the proposed framework to exploit the complementary representation capabilities of different transformer architectures while preserving a unified MTL formulation.

The rationale behind selecting these three transformer architectures is based on their complementary representation capabilities. ViT effectively captures long-range global contextual relationships through full self-attention, facilitating holistic facial representation learning. Swin Transformer introduces hierarchical feature extraction and shifted-window attention, enabling efficient modeling of local facial structures together with global contextual information. DeiT, on the other hand, provides a computationally efficient transformer architecture optimized through knowledge distillation and data-efficient learning. Rather than assuming the superiority of a single architecture, the proposed framework investigates whether these complementary learning characteristics can be effectively integrated into a unified prediction model.

To exploit these complementary representations, two ensemble learning strategies are investigated. The first employs a conventional standard ensemble, where the outputs of the three transformer models are combined using equal contributions. The second introduces a TAWE, in which model contributions are determined independently for age estimation and gender classification according to their validation performance. Consequently, transformer architectures demonstrating superior performance for a specific task contribute more strongly to the final prediction of that task. This task-specific validation-based weighting strategy allows the ensemble to assign different contributions to the transformer models according to their validation performance for each prediction task, thereby exploiting their complementary characteristics without introducing additional trainable fusion parameters.

**Figure 2:**
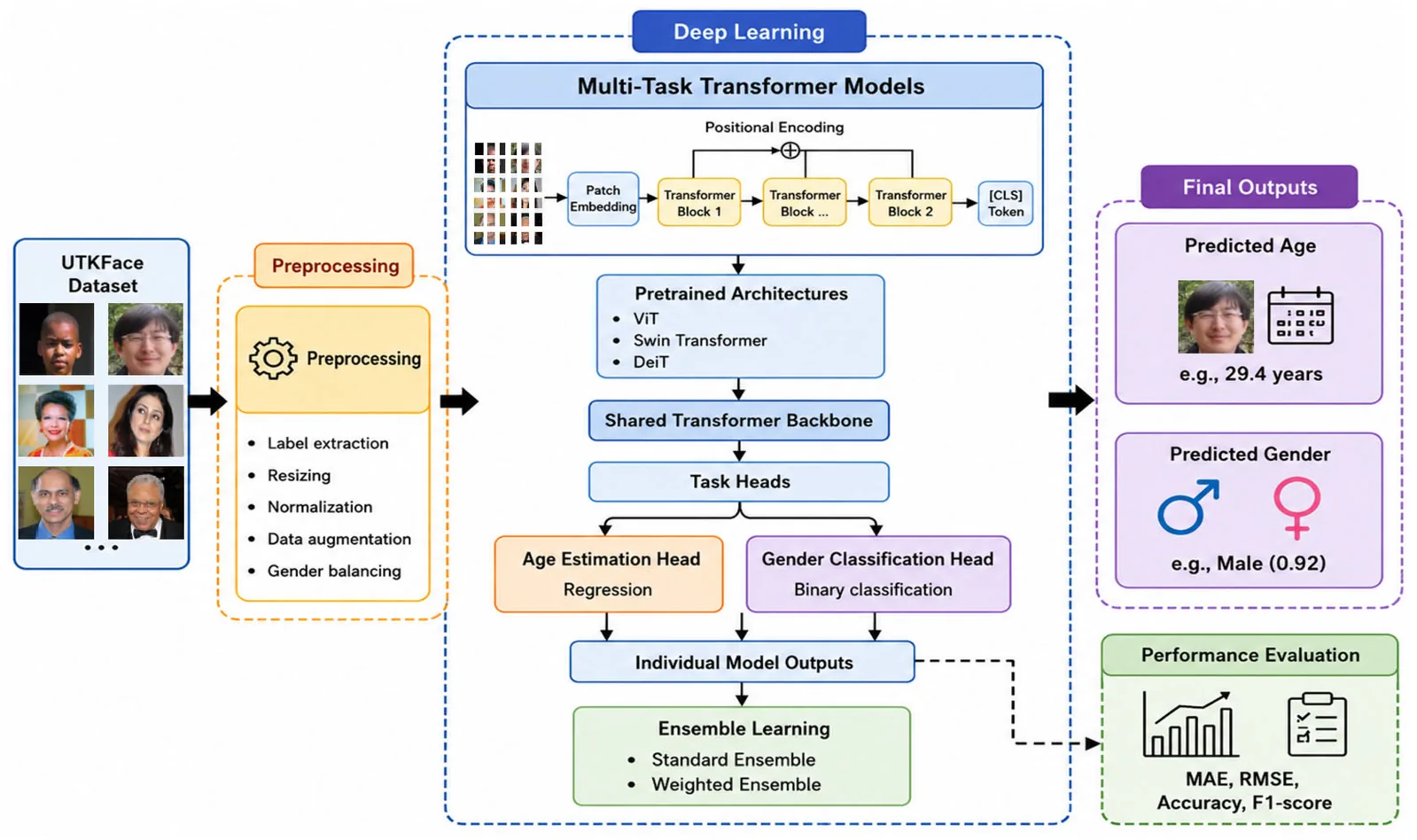
Overall architecture of the proposed transformer-based MTL framework integrating complementary transformer backbones through the proposed TAWE strategy.

Following the conceptual comparison presented in Figure 1, the detailed architecture and workflow of the proposed TAWE-based MTL framework are illustrated in Figure 2. The framework first preprocesses facial images before feeding them into three complementary transformer-based multi-task models. Their task-specific predictions are subsequently integrated using either standard ensemble learning or the proposed TAWE strategy to generate the final age estimation and gender classification results. Facial images are first subjected to preprocessing operations and subsequently provided as input to three independent transformer-based MTL models. Within each architecture, a shared transformer backbone extracts high-level facial representations that are simultaneously utilized by two task-specific prediction heads, one performing continuous age regression and the other conducting binary gender classification. Finally, predictions generated by the individual transformer models are integrated through standard and TAWE strategies to produce the final demographic estimation results.

Based on the above considerations, the principal contributions of this study can be summarized as follows:

- A unified transformer-based MTL framework is proposed to simultaneously perform facial age estimation and gender classification using shared feature representations and task-specific prediction heads.
- Three complementary transformer architectures (ViT, Swin Transformer, and DeiT) are systematically investigated under an identical MTL setting, enabling a comprehensive comparative analysis of their demographic prediction capabilities.
- A task-aware validation-based weighting strategy, referred to as TAWE, is introduced to assign independent model contributions for age estimation and gender classification based on task-specific validation performance, enabling the ensemble to exploit complementary transformer representations rather than relying on uniform weighting.
- A comprehensive experimental evaluation is conducted on the publicly available UTKFace dataset using regression and classification performance metrics, including MAE, RMSE, Accuracy, and F1-score, providing a detailed assessment of both demographic prediction tasks.
- Experimental results demonstrate that the proposed weighted ensemble achieved the best point estimates among the evaluated ensemble strategies, while providing a statistically significant improvement in gender classification accuracy over equal-weight averaging and maintaining comparable age-estimation performance.

Overall, this study extends existing transformer-based facial analysis research by integrating complementary transformer architectures within a unified MTL framework and introducing a task-aware ensemble mechanism that exploits the strengths of each backbone according to the target prediction task. The proposed framework provides a flexible and extensible approach for simultaneous multi-task facial demographic analysis, while the reported findings remain limited to the evaluated UTKFace setting.

## 2. Materials and Methods

This section describes the experimental framework developed for simultaneous age estimation and gender classification from facial images. The proposed methodology consists of five major stages: dataset preparation, image preprocessing and augmentation, transformer-based MTL, task-aware ensemble learning, and performance evaluation. Initially, facial images are collected and organized into training, validation, and testing subsets while preserving a balanced gender distribution. Subsequently, image preprocessing and augmentation techniques are applied to improve the robustness and generalization capability of the learning models. Three transformer-based MTL architectures, namely ViT, Swin Transformer, and DeiT, are then independently trained using identical experimental settings. Each architecture employs a shared transformer backbone for feature extraction together with two task-specific prediction heads responsible for age estimation and gender classification, respectively. Finally, the outputs of the individual transformer models are integrated using both conventional equal-weight ensemble learning and the proposed TAWE strategy. The overall methodology enables a comprehensive comparison of different transformer architectures while simultaneously investigating the effectiveness of task-specific validation-based ensemble learning for facial demographic analysis.

Unlike conventional single-task learning approaches, the proposed framework jointly learns age estimation and gender classification from a common facial representation space. Since both demographic attributes originate from the same facial appearance and share numerous discriminative visual characteristics, shared representation learning enables the extraction of richer and more generalized facial features while simultaneously reducing redundant computations. Furthermore, integrating multiple transformer architectures allows complementary feature representations to be exploited, improving both prediction robustness and generalization performance under challenging imaging conditions.

### 2.1. Dataset

The experiments were conducted using the UTKFace (Large Scale Face Dataset), one of the most widely adopted benchmark datasets for facial demographic analysis and age estimation research [14]. The dataset contains unconstrained facial images collected from individuals with diverse demographic characteristics, covering a broad range of ages, genders, ethnicities, facial expressions, illumination conditions, head poses, image resolutions, and appearance variations. Owing to these large intra-class and inter-class variations, UTKFace provides a challenging benchmark for evaluating transformer-based facial attribute prediction under diverse imaging conditions.

Each facial image in the UTKFace dataset is annotated through its filename, which encodes four demographic attributes: chronological age, gender, ethnicity, and acquisition timestamp. Although the original dataset provides multiple demographic labels, the present study focuses exclusively on age estimation and gender classification. Consequently, the chronological age label is employed as the target variable for the regression task, whereas the gender label is utilized for binary classification. Ethnicity information is intentionally excluded from the learning process to ensure that the proposed framework concentrates solely on the joint prediction of age and gender.

After removing invalid samples and organizing the dataset, a total of 15,503 facial images were included in the experimental study. To ensure an unbiased evaluation protocol, 3285 images were first reserved as an independent test set and were not involved in any stage of model training or hyperparameter optimization. The remaining 12,218 images constituted the development dataset used for training and validation.

Since an imbalance between male and female samples may bias the optimization process toward the majority class, gender balancing was performed before model training. Specifically, an equal number of male and female samples were retained, resulting in a balanced training pool containing 6109 facial images for each gender category. This balancing strategy enables the learning algorithm to receive comparable supervision from both gender classes and reduces the influence of class imbalance during model optimization.

**Figure 3:**
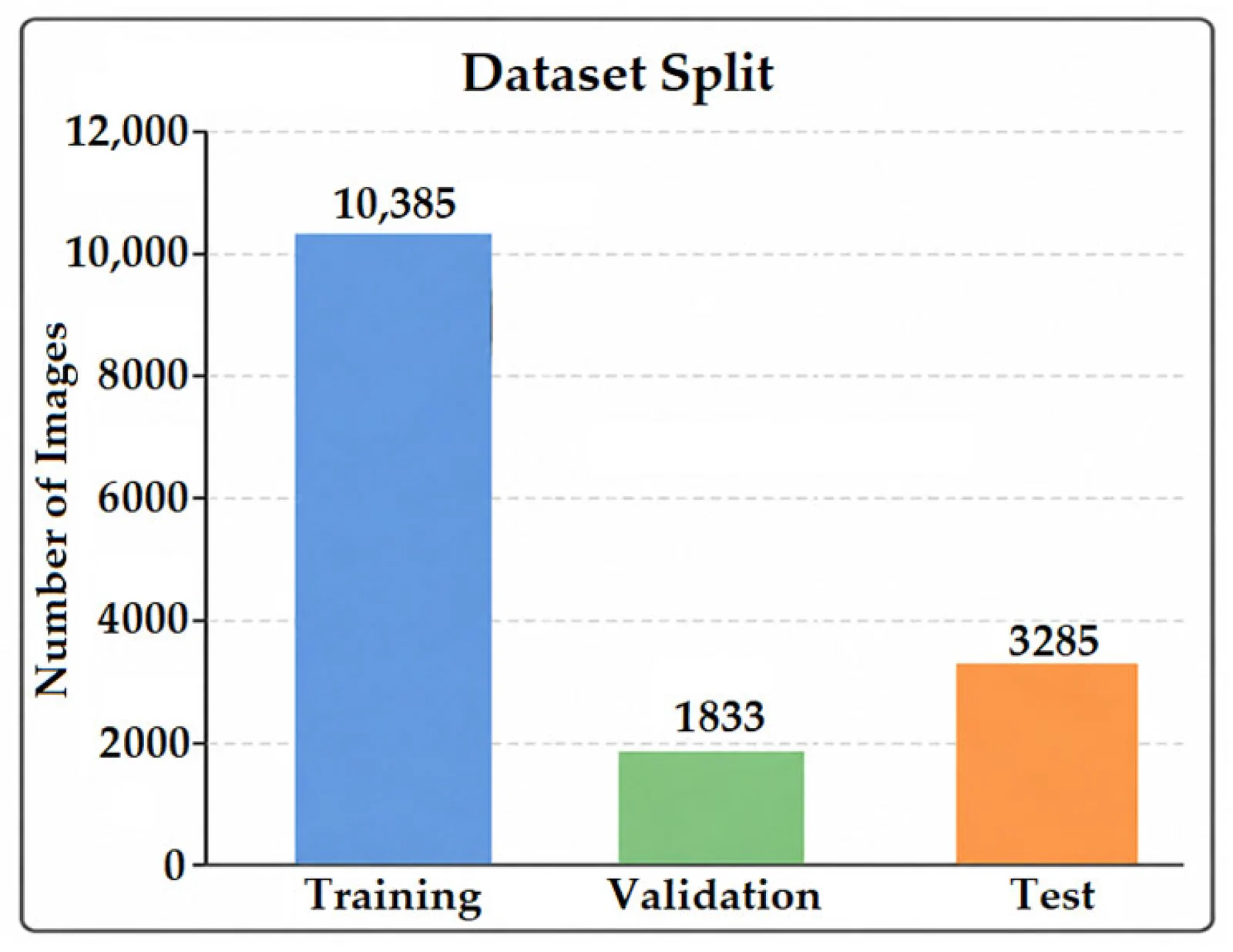
The UTKFace facial image dataset.

Figure 3 presents representative facial images from the UTKFace dataset, illustrating the considerable diversity in age, gender, facial appearance, illumination, pose, and image quality. These variations make demographic prediction substantially more challenging than laboratory-controlled facial datasets and therefore provide a realistic benchmark for evaluating transformer-based facial analysis systems.

The balanced development dataset was subsequently divided into 85% training and 15% validation subsets. Accordingly, the final experimental dataset consisted of 10,385 training images, 1833 validation images, and 3285 testing images, as illustrated in Figure 3. Maintaining identical data partitions throughout all experiments ensures that the comparative analysis among ViT, Swin Transformer, DeiT, and the proposed ensemble strategies remains fair, reproducible, and statistically consistent. For clarity, the final dataset distribution is summarized in Table 2.

### 2.2. Image Preprocessing and Data Augmentation

Before model training, all facial images underwent a standardized preprocessing pipeline to ensure compatibility with the transformer architectures and to reduce the influence of unwanted image variations. First, each image was resized to 224 × 224 pixels, which corresponds to the default input resolution adopted by ViT, Swin Transformer, and DeiT. Using a unified input size guarantees that differences in model performance originate from architectural characteristics rather than inconsistencies in image resolution.

To improve model robustness and reduce overfitting, online data augmentation was applied exclusively during the training stage. Unlike offline augmentation, online augmentation generates transformed samples during mini-batch construction without physically increasing the dataset size. This strategy improves sample diversity while maintaining efficient storage requirements.

The augmentation pipeline consisted of three fundamental transformations:

- Image resizing to ensure consistent spatial dimensions across all transformer models.
- Random horizontal flipping, which enables the model to learn mirror-invariant facial representations and improves robustness to left–right pose variations.
- Color jittering, which randomly modifies image brightness, contrast, saturation, and hue, thereby reducing sensitivity to illumination changes and camera acquisition conditions.

Importantly, these augmentation operations were applied only during training. The validation and test datasets were intentionally left free from random transformations to provide an unbiased estimation of the generalization capability of the trained models. During inference, only deterministic resizing and normalization operations were performed, ensuring that all transformer architectures were evaluated under identical conditions.

Standard image normalization was subsequently applied using the predefined normalization parameters associated with the ImageNet pretrained weights. Employing the same normalization strategy across ViT, Swin Transformer, and DeiT ensures consistent feature distributions and facilitates fair comparisons among the investigated transformer architectures.

### 2.3. Proposed Transformer-Based Multi-Task Learning (MTL) Architecture

The proposed framework is designed to simultaneously perform facial age estimation and gender classification within a unified MTL architecture. Instead of developing two independent deep learning models, a shared feature extraction strategy is adopted to exploit the common visual characteristics underlying both demographic attributes. Since facial geometry, skin texture, craniofacial structure, and age-related morphological patterns contain discriminative information for both age estimation and gender recognition, learning these tasks jointly enables the model to extract richer feature representations while reducing redundant computations.

The proposed framework consists of three independent transformer-based MTL models constructed using ViT, Swin Transformer, and DeiT backbones. Although the internal feature extraction mechanisms of these architectures differ, all models share the same overall learning framework. Each architecture contains three major components:

- A shared transformer backbone responsible for hierarchical facial feature extraction,
- An age regression head that predicts continuous age values,
- A gender classification head that estimates the probability of each gender class.

**Figure 4:**
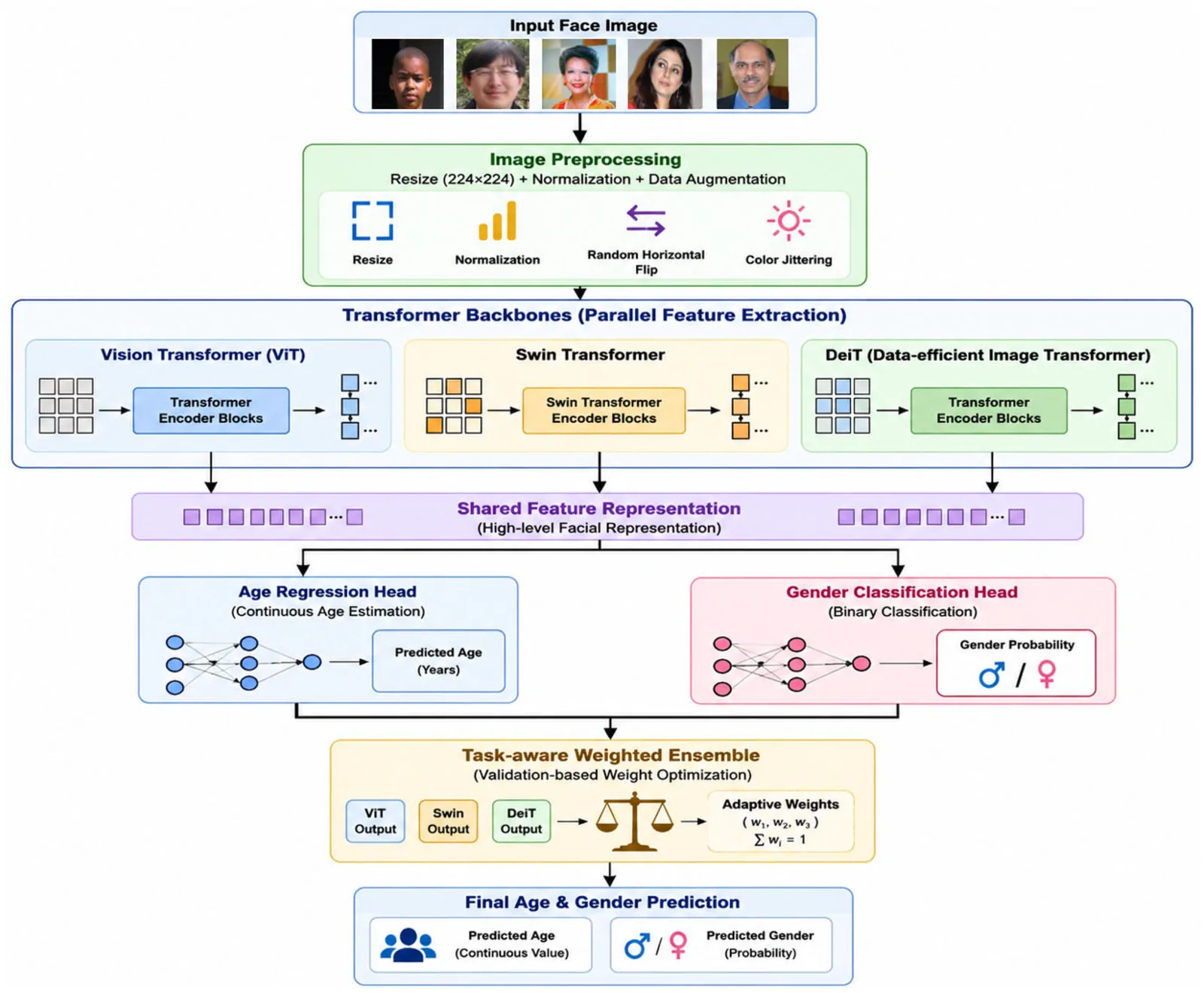
Overall architecture of the proposed transformer-based MTL framework for simultaneous age estimation and gender classification. The framework consists of three complementary transformer backbones (ViT, Swin Transformer, and DeiT), followed by task-specific prediction heads and a task-specific validation-based weighting module for final prediction.

Figure 4 illustrates the overall architecture of the proposed framework.

Initially, an input facial image is resized to 224 × 224 pixels and divided into non-overlapping image patches. The generated patch sequence is processed by the transformer backbone to learn high-level contextual facial representations through successive self-attention operations. Unlike conventional CNNs that gradually enlarge the receptive field using convolution kernels, transformer architectures establish long-range dependencies among all facial regions via the self-attention mechanism, allowing both global facial geometry and local discriminative characteristics to be represented simultaneously.

The extracted deep feature representation is subsequently shared by two independent prediction branches. The first branch performs continuous age estimation through a regression layer, whereas the second branch estimates gender probabilities using a binary classification layer. This shared representation learning strategy enables the network to utilize complementary demographic information while maintaining task-specific prediction capability.

Overall, the proposed framework combines the representation power of transformer architectures with the efficiency of MTL, providing a unified solution for facial demographic analysis.

#### 2.3.1. Shared Transformer Backbone

The shared transformer backbone constitutes the core component of the proposed framework, where high-level facial representations are extracted and subsequently transferred to multiple prediction tasks. Instead of learning independent feature extractors for age estimation and gender classification, the proposed architecture employs a single shared encoder that simultaneously captures the visual information required by both tasks. This design reduces computational redundancy while encouraging the learning of generalized facial representations.

Given an input facial image:

(1) $$I\in R^{H\times W\times C}$$

where *H*, *W*, and *C* denote image height, width, and the number of color channels, respectively, the image is first partitioned into a sequence of fixed-size patches. Assuming a patch size of *P* × *P*, the total number of image patches becomes:

(2) $$N=\frac{H\;W}{P^{2}}$$

Each image patch is flattened and projected into a latent embedding space through a learnable linear projection defined in Equation (3):

(3) $$x_{i}=W_{e}p_{i}+b_{e}$$

where $p_{i}$ denotes the flattened image patch, $W_{e}$ represents the embedding matrix, and $b_{e}$ is the learnable bias vector. Following the original ViT formulation, positional information is incorporated into the embedded patches by adding learnable positional embeddings:

(4) $$z_{0}=\left[x_{cls};x_{1};x_{2};\ldots x_{N}\right]+E_{pos},$$

where $x_{cls}$ denotes the classification token, $E_{pos}$ represents the positional embedding matrix. The embedded sequence is subsequently processed through multiple transformer encoder blocks. Each encoder block consists of two fundamental operations:

- Multi-Head Self-Attention (MHSA)
- Feed-Forward Network (FFN)

together with residual connections and layer normalization. For a given feature representation *X*, the self-attention mechanism is defined as:

(5) $$Q={XW}_{Q},\;\;K={XW}_{K},\;\;V={XW}_{V},$$

where *Q*, *K*, and *V* represent the query, key, and value matrices, respectively. The attention weights are computed by

(6) $$Attention\left(Q,\;K,\;V\right)=Softmax\left(\frac{{QK}^{T}}{\sqrt{d}}\right)V,$$

where d denotes the feature dimension. Unlike convolutional operations that aggregate only local neighborhoods, the self-attention mechanism establishes pairwise relationships among all image patches, enabling the model to capture long-range contextual dependencies distributed across different facial regions.

The output of the attention layer is combined with the input through a residual connection:

(7) $$X^{l+1}=X^{l}+MHSA\left(LN(X^{l})\right),$$

where *LN*(⋅) denotes Layer Normalization. The feature representation is then refined using a feed-forward network,

(8) $$X^{l+2}=X^{l+1}+FFN\left(LN(X^{l+1})\right),$$

This process is repeated throughout multiple transformer encoder blocks, progressively learning increasingly abstract facial representations.

Although the proposed framework employs three different transformer architectures (ViT, Swin Transformer, and DeiT), all of them ultimately produce a high-dimensional feature vector:

(9) $$F\in R^{D}$$

which serves as the shared representation for the subsequent multi-task prediction heads. This shared feature vector simultaneously preserves global facial structure, local texture information, and contextual semantic relationships, thereby providing a comprehensive representation suitable for both age estimation and gender classification.

The shared backbone constitutes the foundation of the proposed MTL framework because it enables efficient knowledge transfer between related demographic prediction tasks while reducing model complexity compared with independently trained networks. Furthermore, learning a common feature representation facilitates the subsequent task-aware ensemble strategy by ensuring that complementary information extracted by different transformer architectures can be effectively integrated during the final prediction stage [15].

#### 2.3.2. Age Regression Head

Following the shared transformer backbone, the extracted high-level facial representation is forwarded to the Age Regression Head, which is specifically designed to estimate the chronological age of an individual from facial appearance. Unlike conventional classification problems, age estimation is formulated as a continuous regression task, where the objective is to predict an age value that is numerically as close as possible to the ground-truth label. This formulation enables the model to preserve the natural ordering among age values and avoids the information loss associated with discretizing ages into predefined categories.

Let the shared feature vector generated by the transformer backbone be denoted as $F\in R^{D}$. Where *D* represents the feature dimension. The feature vector is first processed through a fully connected transformation to learn task-specific representations for age prediction:

(10) $$F_{age}=\phi (W_{age}F+b_{age}),$$

where $W_{age}$ and $b_{age}$ denote the learnable weight matrix and bias vector, respectively, while *ϕ*(⋅) represents the nonlinear activation function. To reduce overfitting and improve generalization capability, a dropout layer is incorporated before the final regression layer. The age prediction is then obtained through a linear regression unit:

(11) $${\hat{y}}_{age}=({W_{r}F}_{age}F+b_{r}),$$

where ${\hat{y}}_{age}$ denotes the predicted age, $W_{r}$ represents the regression weight matrix, and $b_{r}$ denotes the regression bias. Unlike classification-based age estimation methods, the proposed regression formulation directly predicts continuous age values without requiring predefined age intervals or ordinal age categories. Consequently, the model is capable of learning subtle age-related facial changes that occur continuously throughout the human aging process.

During optimization, the discrepancy between the predicted age and the corresponding ground-truth age is minimized using the Mean Squared Error (MSE) loss:

(12) $$L_{age}=\;\frac{1}{N}\sum_{i=1}^{N}{\left(y_{i}-{\hat{y}}_{i}\right)}^{2},$$

where *N* denotes the batch size, $y_{i}$ represents the ground-truth chronological age, and ${\hat{y}}_{i}$ is the predicted age. Although the model is optimized using the MSE objective because of its favorable optimization properties, its prediction performance is evaluated using both Mean Absolute Error (MAE) and Root Mean Squared Error (RMSE), providing complementary information regarding prediction accuracy and robustness.

The dedicated regression head enables the network to specialize in learning age-sensitive facial characteristics while simultaneously benefiting from the generalized facial representations extracted by the shared transformer backbone. As a result, both common demographic information and age-specific discriminative patterns contribute to the final age estimation process.

#### 2.3.3. Gender Classification Head

In parallel with the age regression branch, the proposed framework includes an independent Gender Classification Head responsible for predicting the binary gender label defined in the UTKFace dataset from facial images. Since gender prediction is formulated as a binary classification problem, the objective of this branch is to estimate the posterior probability of each gender class while sharing the same high-level facial representation learned by the transformer backbone.

Using the shared feature vector *F*, a task-specific gender representation is first generated through a fully connected transformation:

(13) $$F_{gender}=\;\phi (W_{gender}F+b_{gender}),$$

where $W_{gender}$ and $b_{gender}$ represent learnable model parameters. The obtained representation is subsequently transformed into class logits:

(14) $$z=\;W_{c}F_{gender}+b_{c}$$

where *z* denotes the unnormalized class scores. The probability of each gender class is computed through the Softmax activation:

(15) $$P\left(c_{i}\right)=\frac{e^{z_{i}}}{\sum_{j=1}^{C}e^{z_{i}}}$$

where *C* = 2 denotes the number of gender classes, $P\left(c_{i}\right)$ represents the posterior probability of class *i*. The predicted gender label is determined according to

(16) $$\hat{c}=\arg\underset{i}{\max}P\left(c_{i}\right).$$

To optimize the gender classification branch, the Cross-Entropy Loss is employed:

(17) $$L_{gender}=\;-\frac{1}{N}\sum_{i=1}^{N}\sum_{c=1}^{C}y_{ic}\log P\left(c_{i}\right),$$

where $y_{ic}$ denotes the one-hot encoded ground-truth label, $P\left(c_{i}\right)$ is the predicted class probability.

Cross-entropy optimization encourages the model to maximize the posterior probability of the correct gender class while minimizing incorrect predictions. Owing to the balanced training dataset introduced during the preprocessing stage, both gender categories contribute equally to parameter optimization, thereby reducing potential prediction bias toward the majority class.

Unlike independently trained gender classifiers, the proposed gender prediction head benefits from facial representations jointly optimized for both demographic tasks. The shared backbone allows gender classification to exploit age-related facial characteristics when beneficial while maintaining sufficient task-specific flexibility through its dedicated classification layers. Consequently, the proposed multi-task design enhances both feature efficiency and prediction robustness under unconstrained facial imaging conditions.

#### 2.3.4. Multi-Task Loss Function

The proposed framework simultaneously optimizes two correlated learning objectives, namely continuous age estimation and binary gender classification, within a unified MTL paradigm. Instead of training two independent neural networks, the proposed architecture shares a common transformer backbone while employing dedicated prediction heads for each task. Consequently, model parameters are updated according to a joint optimization objective that balances the contributions of both regression and classification tasks.

MTL improves the generalization capability of deep neural networks by enabling related tasks to exchange useful information during feature learning. Since age estimation and gender classification are derived from the same facial image and share numerous discriminative visual characteristics, jointly optimizing these tasks encourages the backbone network to learn richer and more representative facial features than conventional single-task optimization.

Let the training dataset be represented as

(18) $$D=\{(x_{i},y_{i}^{age},y_{i}^{gender}){\}}_{i=1}^{N},$$

where $x_{i}$ denotes the input facial image, $y_{i}^{age}$ is the ground-truth age, $y_{i}^{gender}$ represents the corresponding gender label, and *N* denotes the total number of training samples. During forward propagation, the shared transformer backbone extracts a latent facial representation:

(19) $$F=f_{\theta }(x),$$

where $f_{\theta }$(⋅) denotes the shared transformer encoder, *θ* represents the learnable backbone parameters. The extracted feature representation is simultaneously transferred to the two task-specific prediction heads:

(20) $${\hat{y}}^{age}=g_{age}\left(F\right),\;\;{\hat{y}}^{gender}=g_{gender}\left(F\right),$$

where $g_{age}$(⋅) denotes the regression head, $g_{gender}$(⋅) denotes the classification head. Accordingly, two independent task losses are computed. The regression branch minimizes the MSE:

(21) $$L_{age}=\;\frac{1}{N}\sum_{i=1}^{N}{(y}_{i}^{age}-{{\hat{y}}_{i}^{age})}^{2}.$$

Meanwhile, the classification branch minimizes the categorical cross-entropy loss:

(22) Lgender= −1N∑i=1N∑c=1Cyiclog p^ic,

where *C* denotes the number of gender classes, ${\hat{p}}_{ic}$ is the predicted probability of class *c*. Rather than minimizing these objectives independently, both losses are jointly optimized through a weighted linear combination:

(23) $$L_{total}=\lambda_{age}L_{age}+\lambda_{gender}L_{gender},$$

where $\lambda_{age}$ and $\lambda_{gender}$ represent the task-balancing coefficients. Throughout this study, equal task importance is assumed:

(24) $$\lambda_{age}=\lambda_{gender}=0.5,$$

ensuring that neither task dominates the optimization process during training. The overall parameter optimization objective can therefore be expressed as:

(25) $$\theta^{*}=\arg\underset{\theta }{\min}L_{total}.$$

Model parameters are updated iteratively using backpropagation,

(26) $$\theta_{t+1}=\theta_{t}-\eta \nabla_{\theta }L_{total},$$

where *η* denotes the learning rate, $\nabla_{\theta }L_{total}$ represents the gradient of the joint loss function. This joint optimization strategy allows gradients generated by both demographic prediction tasks to simultaneously influence the shared transformer backbone. Consequently, feature representations become more informative and generalized because they encode facial characteristics beneficial to both age estimation and gender classification.

An additional advantage of the proposed optimization strategy is its ability to reduce overfitting. Since both tasks constrain the shared feature space from different perspectives, the network is encouraged to learn generalized facial representations instead of task-specific noise. This collaborative learning mechanism improves model robustness, particularly under challenging imaging conditions involving pose variation, illumination changes, facial expressions, and age-related appearance diversity.

Furthermore, employing separate prediction heads together with a shared optimization objective provides an effective balance between knowledge sharing and task specialization. While the transformer backbone learns common facial representations, each prediction head remains free to optimize features that are uniquely relevant to its corresponding task. This design significantly improves learning efficiency while maintaining sufficient flexibility for heterogeneous prediction objectives.

Overall, the proposed joint loss formulation constitutes the core optimization mechanism of the proposed multi-task transformer framework and provides the mathematical foundation for simultaneously learning continuous age estimation and binary gender classification within a unified deep learning architecture.

#### 2.3.5. Transfer Learning Strategy

Training transformer models entirely from randomly initialized parameters generally requires millions of annotated images and extensive computational resources. However, facial demographic datasets are considerably smaller than large-scale image recognition datasets, making end-to-end training susceptible to overfitting. To overcome this limitation, the proposed framework adopts a transfer learning strategy in which all transformer backbones are initialized using weights pretrained on the ImageNet dataset.

ImageNet pretraining enables the transformer encoder to learn generic visual representations such as edges, textures, shapes, and semantic object structures before being adapted to the facial demographic analysis task. These pretrained representations significantly accelerate convergence and improve optimization stability, especially during the early training epochs.

In the proposed framework, the pretrained transformer parameters serve as the initialization of the shared backbone, whereas the age regression head and gender classification head are randomly initialized. During fine-tuning, all network parameters are updated simultaneously through backpropagation, allowing the pretrained visual representations to gradually adapt to facial age estimation and gender classification.

To prevent catastrophic forgetting while preserving the discriminative capability of pretrained features, a relatively small learning rate is employed during fine-tuning. This strategy enables the backbone to retain generic visual knowledge acquired from large-scale image classification while progressively learning task-specific facial representations required for demographic prediction.

Unlike feature extraction approaches that freeze pretrained backbones, the proposed end-to-end fine-tuning strategy allows all transformer parameters to participate in optimization. Consequently, both low-level visual representations and high-level semantic features are jointly adapted to the target dataset, leading to improved feature discrimination and enhanced generalization performance.

The same transfer learning protocol is applied consistently to the ViT, Swin Transformer, and DeiT architectures. Employing identical initialization and optimization settings ensures that the comparative evaluation reflects architectural differences rather than discrepancies in the training procedure, thereby providing a fair and reproducible assessment of the proposed framework.

Although all three transformer architectures share the same MTL framework and optimization strategy, they differ substantially in terms of architectural design, model complexity, and feature extraction mechanisms. These differences may influence both computational efficiency and predictive performance. The principal architectural characteristics of the pretrained transformer backbones employed in this study are summarized in Table 3. Presenting these specifications facilitates a more comprehensive interpretation of the comparative experimental results discussed in the subsequent sections.

### 2.4. Ensemble Learning Strategy

Although transformer-based architectures have demonstrated remarkable success in facial analysis tasks, individual models often exhibit different strengths depending on the target prediction problem. For example, one architecture may generate more accurate age estimates by capturing subtle facial texture variations, whereas another may achieve superior gender classification performance by learning more discriminative global facial structures. Consequently, relying on a single transformer architecture may not fully exploit the complementary representation capabilities available across different models.

To address this limitation, the proposed framework integrates the outputs of three independently trained multi-task transformer models ViT, Swin Transformer, and DeiT through an ensemble learning strategy. Instead of assuming that a single architecture is universally optimal, the proposed ensemble combines the complementary predictions of all transformer backbones to exploit their task-specific strengths.

**Figure 5:**
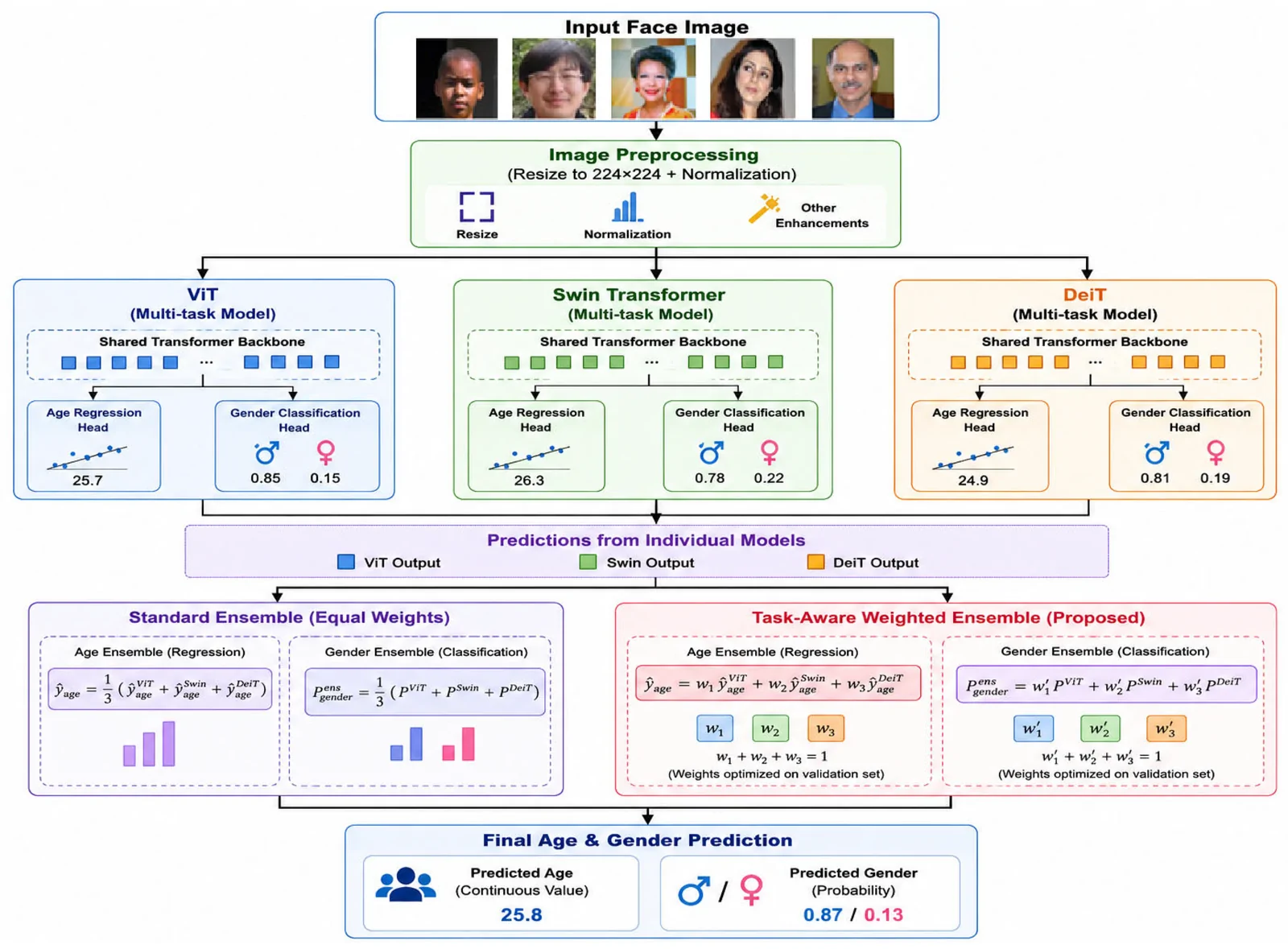
Overview of the proposed Ensemble learning strategy. Predictions generated by the three transformer-based multi-task models are integrated using both standard equal-weight averaging and the proposed TAWE mechanism to obtain the final age estimation and gender classification results.

The ensemble framework is implemented in two consecutive stages. First, a standard ensemble is constructed by assigning equal importance to the predictions of all transformer models. Subsequently, a TAWE is proposed, where model contributions are determined independently for age estimation and gender classification according to their validation performance. The overall ensemble framework is illustrated in Figure 5.

#### 2.4.1. Standard Ensemble

The first ensemble strategy employs conventional equal-weight averaging. Since three transformer models are independently trained using identical data partitions, each model produces an independent prediction for both demographic tasks.

For age estimation, the final prediction is obtained by averaging the regression outputs of the three transformer models:

(27) $${\hat{y}}^{age}=\frac{1}{3}({\hat{y}}_{ViT}+{\hat{y}}_{Swin}+{\hat{y}}_{DeiT})$$

This averaging operation reduces the influence of prediction errors originating from individual models and generally improves regression stability.

For gender classification, each transformer produces posterior probabilities after the Softmax layer $P_{ViT},\;P_{Swin},P_{DeiT}$.

The ensemble probability is computed as

(28) $$P_{ensemble}=\frac{1}{3}\left(P_{ViT}+P_{Swin}+P_{DeiT}\right).$$

The predicted gender label is then determined by

(29) $$\hat{c}=\arg\max P_{ensemble}.$$

The standard ensemble assumes that all transformer architectures contribute equally to the final prediction. Although this simple strategy generally improves robustness compared with individual models, it ignores the fact that different transformer architectures may exhibit task-dependent performance characteristics.

#### 2.4.2. Proposed Task-Aware Weighted Ensemble (TAWE)

Experimental observations indicate that the three transformer architectures do not contribute equally to both demographic prediction tasks. Specifically, Swin Transformer achieves lower regression error in age estimation, whereas ViT provides superior gender classification accuracy. Therefore, assigning identical weights to all models may prevent the ensemble from fully exploiting their complementary strengths.

To overcome this limitation, this study proposes a TAWE in which independent weighting coefficients are assigned to each transformer architecture for each prediction task. For age estimation, the weighted prediction is formulated as:

(30) $${\hat{y}}_{age}=\omega_{1}{\hat{y}}_{ViT}+\omega_{2}{\hat{y}}_{Swin}+\omega_{3}{\hat{y}}_{DeiT},$$

subject to $\omega_{1}+\omega_{2}+\omega_{3}=1,$ and $\omega_{i}\geq 0.$ Similarly, the weighted gender probability is computed as:

(31) $$P_{gender}=\omega_{1}^{\prime }P_{ViT}+\omega_{2}^{\prime }P_{Swin}+\omega_{3}^{\prime }P_{DeiT},$$

where $\omega_{1}^{\prime }+\omega_{2}^{\prime }+\omega_{3}^{\prime }=1.$ Unlike the standard ensemble, the proposed framework allows different transformer architectures to contribute with different importance according to the prediction task.

The task-specific weighting coefficients are selected using the validation dataset. Candidate weight combinations satisfying $\omega_{ViT}+\omega_{Swin}+\omega_{DeiT}=1$ are evaluated. For age estimation, the combination yielding the lowest validation MAE is selected, whereas for gender classification, the combination yielding the highest validation accuracy is selected. Once selected, these task-specific weights remain fixed during test-time inference and are applied uniformly across all test samples. Thus, TAWE does not perform sample-wise or input-dependent dynamic weighting. Consequently, two independent weight vectors are obtained:

(32) $$W_{age}=\left[\omega_{1},\omega_{2},\omega_{3}\right],\;{\;W}_{gender}=\left[\omega_{1}^{\prime },\omega_{2}^{\prime },\omega_{3}^{\prime }\right].$$

This task-dependent optimization enables each transformer architecture to contribute according to its strongest prediction capability rather than enforcing identical participation across heterogeneous tasks.

Compared with equal-weight averaging, the proposed weighting strategy offers two important advantages. First, it reduces the influence of comparatively weaker models on a particular task, thereby improving prediction accuracy. Second, it preserves complementary information extracted by different transformer architectures while providing task-specific decision fusion.

The complete inference procedure of the proposed ensemble strategy can be summarized as follows:

- The input facial image is simultaneously processed by the ViT, Swin Transformer, and DeiT MTL models.
- Each model independently predicts both age and gender.
- The individual outputs are combined using either equal-weight averaging or the proposed TAWE.
- The integrated prediction is returned as the final age estimation and gender classification result.

Since the weighting coefficients are optimized independently for each demographic task, the proposed framework effectively exploits the complementary learning characteristics of different transformer architectures. This task-specific validation-based integration provides a simple fusion mechanism without introducing additional trainable parameters or substantial computational overhead during inference.

The proposed TAWE was designed based on the observation that different transformer architectures exhibit complementary representation capabilities for different facial demographic tasks. As demonstrated by the individual model evaluations, Swin Transformer consistently achieved lower age estimation errors, whereas ViT provided superior gender classification performance. These findings indicate that assigning identical contributions to all models, regardless of the prediction task, may prevent the ensemble from fully exploiting the strengths of individual transformer architectures.

Instead of employing a fixed equal-weight fusion strategy, TAWE determines independent model contributions for each prediction task according to their validation performance. This validation-driven weighting mechanism allows the ensemble to assign task-specific contributions without introducing additional trainable parameters or increasing optimization complexity. Consequently, the proposed framework preserves the simplicity and reproducibility of conventional ensemble learning while enabling task-specific adaptive model fusion.

The task-specific weights were determined independently on the validation set by optimizing the objective function associated with each prediction task. The resulting weights were 0.25, 0.55, and 0.20 for the ViT, Swin Transformer, and DeiT models, respectively, in age estimation, reflecting the superior regression performance of the Swin Transformer. For gender classification, the optimized weights were 0.55, 0.25, and 0.20 for ViT, Swin Transformer, and DeiT, respectively, assigning greater influence to the ViT model due to its higher validation accuracy. The DeiT model retained a smaller yet non-zero contribution in both tasks, providing complementary predictive information without dominating the final decision.

Alternative ensemble strategies, such as stacking, learnable gating networks, uncertainty-based weighting, or mixture-of-experts architectures, can potentially provide more flexible task-specific validation-based fusion mechanisms. However, these approaches require additional optimization procedures, larger training datasets, and increased computational complexity, which may introduce a higher risk of overfitting, particularly when the available facial dataset is relatively limited. In contrast, the proposed validation-based weighting strategy provides a transparent and computationally efficient solution that directly reflects the empirical strengths of each transformer architecture for each prediction task.

Therefore, rather than proposing a highly parameterized fusion network, this study investigates whether a simple yet task-specific validation-based weighting strategy is sufficient to exploit complementary transformer representations for simultaneous age estimation and gender classification. TAWE achieved the best point estimates among the evaluated ensemble strategies by leveraging the task-specific strengths of the transformer backbones, while the improvement in age estimation over the equal-weight ensemble remained modest and was not statistically significant.

To further justify the proposed TAWE, Table 4 presents a conceptual comparison between the adopted validation-based weighting strategy and several representative ensemble fusion approaches commonly used in deep learning. Unlike learnable fusion methods that introduce additional trainable parameters and optimization complexity, the proposed strategy preserves architectural simplicity while enabling task-specific model contributions based on validation performance.

Overall, the proposed TAWE constitutes the final decision-making stage of the proposed facial demographic analysis framework. Unlike conventional equal-weight averaging, the proposed strategy explicitly acknowledges that different transformer architectures are not equally effective for all prediction tasks. By assigning task-specific contributions according to validation performance, TAWE preserves the complementary strengths of each backbone while maintaining a lightweight and fully interpretable fusion mechanism. Consequently, the proposed framework achieves improved prediction performance without introducing additional trainable parameters or increasing the computational complexity of the ensemble model.

### 2.5. Experimental Settings

To ensure a fair and reproducible comparison, all transformer-based MTL models were trained and evaluated under identical experimental conditions. The ViT, Swin Transformer, and DeiT architectures were implemented using the PyTorch deep learning framework and initialized with ImageNet-pretrained weights. Throughout the experiments, identical data partitions, preprocessing operations, optimization strategies, and evaluation protocols were employed for all models to eliminate potential sources of experimental bias.

All input facial images were resized to 224 × 224 pixels, which corresponds to the default input resolution of the pretrained transformer architectures. During the training stage, online data augmentation was applied through random horizontal flipping and color jittering to improve model robustness and reduce overfitting. Validation and testing images were processed only by deterministic resizing and normalization, ensuring unbiased performance evaluation.

The proposed models were optimized using the AdamW optimizer, which has become one of the standard optimization algorithms for transformer-based vision models due to its effective handling of weight decay and stable convergence characteristics [16]. The initial learning rate was set to 1 × 10^−4^, while the weight decay coefficient was fixed at 1 × 10^−4^ to improve generalization performance. Training was performed using a mini-batch size of 32, and all models were trained for 30 epochs. Model parameters corresponding to the lowest validation loss were retained for the final evaluation.

To prevent unnecessary optimization after convergence, an Early Stopping strategy was employed based on the validation loss. Training was terminated whenever the validation performance failed to improve for a predefined number of consecutive epochs. This strategy effectively reduced the risk of overfitting while preserving the best-performing model parameters.

The complete set of implementation details, computational environment, and training hyperparameters adopted throughout the experiments is summarized in Table 5. The same hyperparameter configuration was consistently applied to the ViT, Swin Transformer, and DeiT architectures. Consequently, any observed performance differences can be attributed primarily to architectural characteristics rather than discrepancies in optimization settings or training procedures.

Following the training of the individual transformer models, two ensemble learning strategies were evaluated. The first employed a conventional equal-weight averaging scheme, whereas the second utilized the proposed TAWE. The weighting coefficients of the proposed ensemble were determined exclusively using the validation dataset and subsequently fixed during testing to avoid information leakage from the independent test set.

As shown in Table 5, identical implementation settings were maintained throughout all experiments to ensure the reproducibility and fairness of the comparative evaluation. By employing the same optimization strategy, hyperparameter configuration, computational environment, and training protocol for all transformer architectures, the experimental results primarily reflect the intrinsic characteristics of the investigated models rather than differences in the training process.

All experiments were performed on a workstation equipped with an Intel Core i7 processor (4.70 GHz) (Intel, Santa Clara, CA, USA), 32 GB RAM, and an NVIDIA GeForce RTX 4060 GPU (8 GB GDDR6) (Nvidia, Santa Clara, CA, USA) running Windows 11 Pro (64-bit).

### 2.6. Performance Evaluation Metrics

To comprehensively evaluate the effectiveness of the proposed framework, separate evaluation metrics were employed for the regression and classification tasks. Since age estimation and gender classification involve fundamentally different prediction objectives, each task was assessed using performance measures specifically designed for its corresponding learning problem.

For the age estimation task, prediction accuracy was quantified using the MAE and the RMSE. MAE measures the average absolute deviation between the predicted and ground-truth ages and is defined as

(33) $$MAE=\frac{1}{N}\sum_{i=1}^{N}\left|y_{i}-{\hat{y}}_{i}\right|,$$

where *N* denotes the total number of test samples, $y_{i}$ is the ground-truth age, and ${\hat{y}}_{i}$ represents the predicted age.

The RMSE metric, which assigns greater penalties to large prediction errors, is calculated as

(34) $$RMSE=\sqrt{\frac{1}{N}\sum_{i=1}^{N}{\left(y_{i}-{\hat{y}}_{i}\right)}^{2}},$$

A lower value of MAE or RMSE indicates better regression performance.

For gender classification, prediction performance was evaluated using Accuracy, Precision, Recall, and F1-score. Accuracy represents the proportion of correctly classified samples and is defined as

(35) $$Accuracy=\frac{TP+TN}{TP+TN+FP+FN}$$

where *TP*, *TN*, *FP*, and *FN* denote the numbers of true positives, true negatives, false positives, and false negatives, respectively.

Precision and Recall are computed as

(36) $$Precision=\frac{TP}{TP+FP},\;\;\;\;\;\;Recall=\frac{TP}{TP+FN}$$

The harmonic mean of Precision and Recall yields the F1-score,

(37) $$F1=2\cdot \frac{Precision\times Recall}{Precision+Recall}$$

These metrics provide complementary perspectives on classification performance, particularly in applications involving potential class imbalance.

To ensure objective comparisons, all evaluation metrics were computed using the independent test dataset. Furthermore, identical evaluation criteria were applied to the individual transformer models, the standard ensemble, and the proposed TAWE, enabling a consistent and statistically fair performance comparison across all investigated approaches.

## 3. Results and Discussion

This section presents a comprehensive experimental evaluation of the proposed transformer-based MTL framework for simultaneous age estimation and gender classification. First, the predictive performance of the three individual transformer architectures—ViT, Swin Transformer, and DeiT—is analyzed using the predefined regression and classification metrics. Subsequently, the effectiveness of the proposed ensemble learning strategies is investigated through a comparative analysis of standard equal-weight averaging and the proposed TAWE. Finally, the experimental findings are interpreted from both quantitative and qualitative perspectives, highlighting the influence of architectural characteristics on demographic prediction performance, discussing the proposed ensemble framework, and providing contextual comparison with representative studies reported in the literature.

### 3.1. Performance of Individual Transformer Models

The first set of experiments was conducted to evaluate the performance of the three transformer-based MTL models before applying ensemble learning. The objective of this analysis was to investigate the individual capabilities of ViT, Swin Transformer, and DeiT for simultaneous age estimation and gender classification under identical training conditions. All models were trained using the same training, validation, and test partitions, identical preprocessing procedures, and the same optimization strategy described in Section 2. This experimental design ensures that the observed performance differences primarily reflect the intrinsic architectural characteristics of the transformer backbones rather than variations in the training process.

The quantitative performance of the individual transformer models is summarized in Table 6. For the age estimation task, MAE and RMSE were adopted to evaluate regression performance, whereas Accuracy, Precision, Recall, and F1-score were used to assess gender classification.

As shown in Table 6, each transformer architecture exhibits distinct strengths depending on the prediction task. Among the evaluated models, Swin Transformer achieved the best age estimation performance, obtaining the lowest MAE (5.1496) and RMSE (6.7717) values. These results indicate that the hierarchical feature extraction mechanism and shifted-window self-attention employed by Swin Transformer are particularly effective in capturing subtle age-related facial characteristics. Unlike conventional global attention mechanisms, the hierarchical design enables the network to simultaneously exploit local texture information and broader facial structures, which are both essential for accurate age prediction.

In contrast, ViT achieved the highest gender classification performance, yielding an accuracy of 94.58%, a precision of 95.17%, a recall of 94.62%, and an F1-score of 94.89%. The superior classification performance of ViT can be attributed to its global self-attention mechanism, which effectively models long-range dependencies across the entire facial image. Since gender classification relies more heavily on holistic facial morphology than localized appearance variations, the global contextual representation learned by ViT provides a significant advantage for this task.

The DeiT model achieved competitive results for both regression and classification tasks but consistently performed below the ViT and Swin Transformer models. Specifically, DeiT produced an MAE of 5.3807, an RMSE of 7.0764, an accuracy of 91.75%, and an F1-score of 92.22%. Although its overall predictive performance was comparatively lower, DeiT exhibited stable convergence characteristics throughout the training process, suggesting that it still learns meaningful facial representations despite its relatively lower predictive capability.

**Figure 6:**
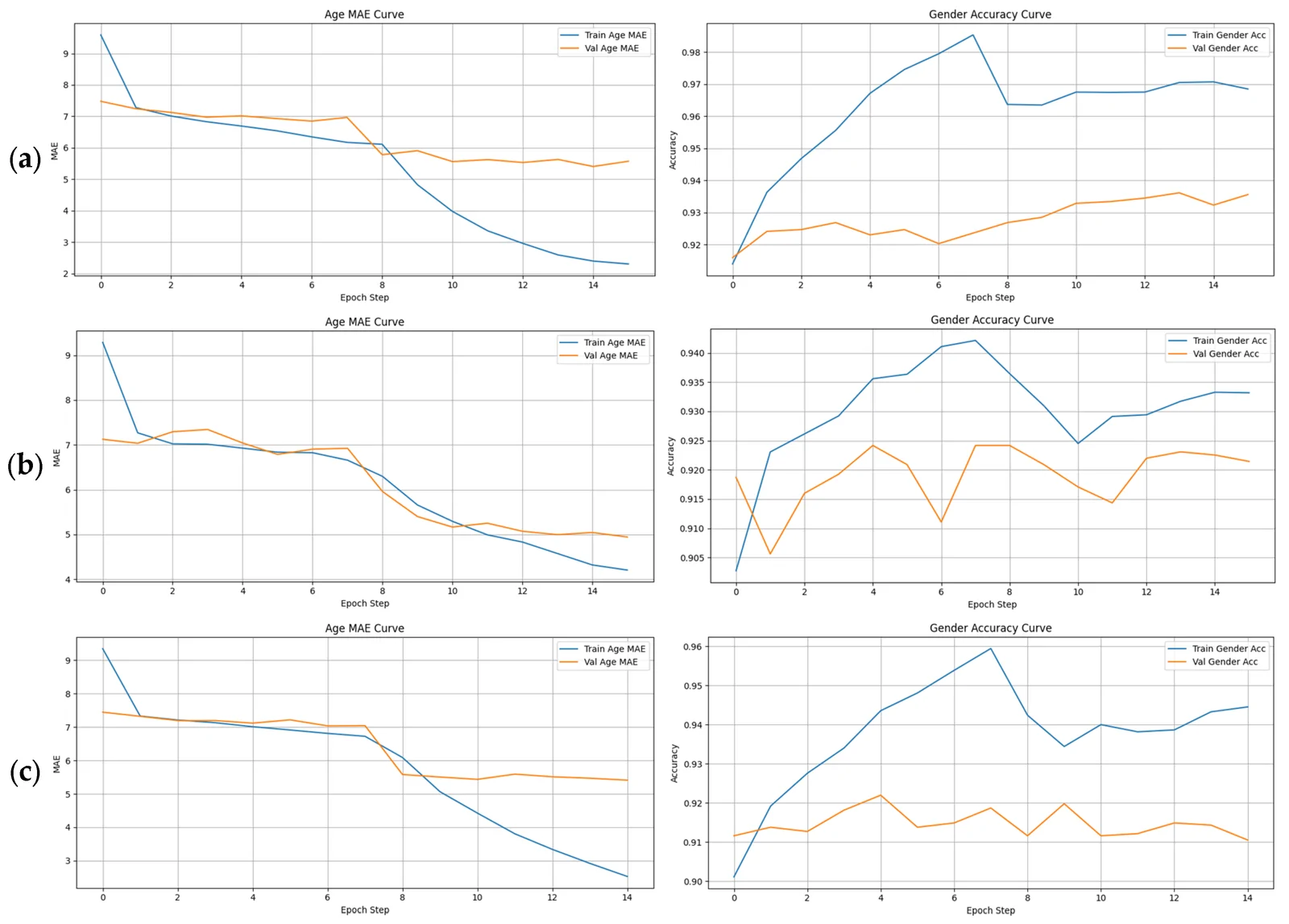
Learning curves showing the evolution of age estimation error (MAE) and gender classification accuracy during training for the individual transformer-based MTL models: (**a**) ViT, (**b**) Swin, and (**c**) DeiT.

To further analyze the optimization behavior of the individual transformer architectures, the learning curves obtained during training are presented in Figure 6. Figure 6 simultaneously illustrates the evolution of the age estimation error (MAE) and gender classification accuracy throughout the training process for each model. These curves provide additional insight into model convergence and training stability beyond the final evaluation metrics reported in Table 6.

As illustrated in Figure 6, all three transformer architectures exhibit stable convergence behavior throughout the training process without evident signs of overfitting. The age estimation error decreases rapidly during the initial epochs before gradually approaching convergence, whereas the gender classification accuracy increases steadily and stabilizes toward the end of training. Among the evaluated models, Swin Transformer consistently maintains the lowest MAE values across most training epochs, confirming its superior capability for learning age-related facial characteristics. Conversely, ViT achieves the highest gender classification accuracy throughout the optimization process, demonstrating the effectiveness of global self-attention for capturing gender-discriminative facial representations. Although DeiT converges slightly more slowly than the other architectures, its learning curves remain smooth and stable, indicating reliable optimization under the proposed MTL framework.

**Figure 7:**
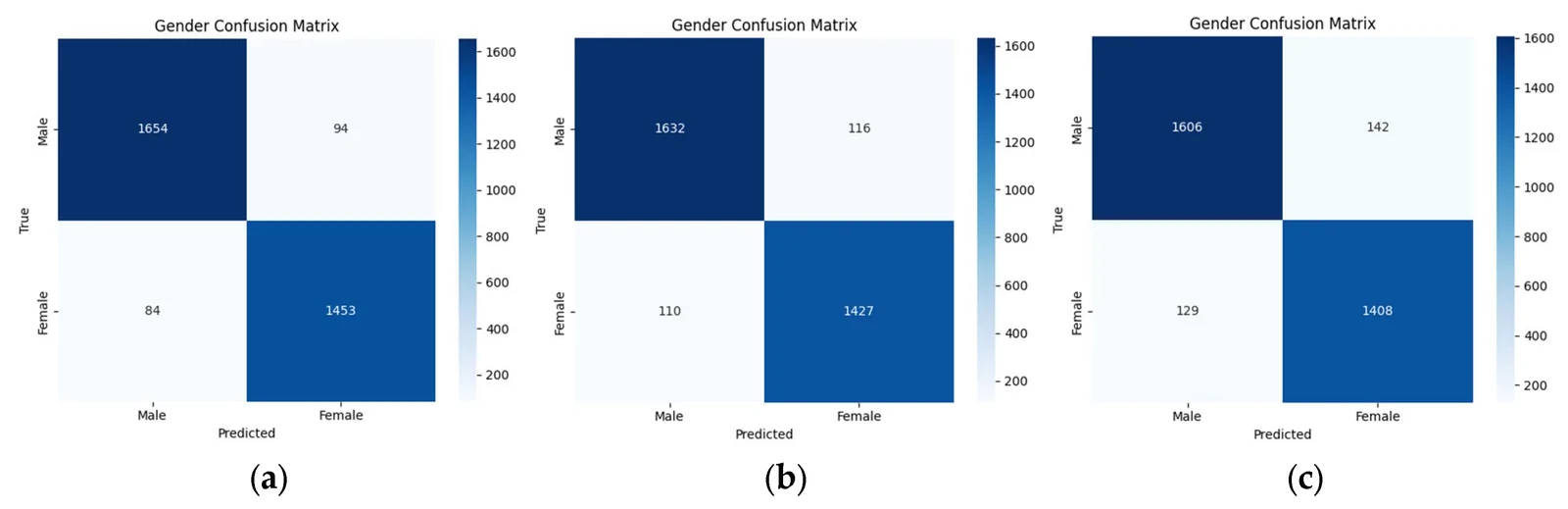
Confusion matrices of the individual transformer-based MTL models for gender classification: (**a**) ViT, (**b**) Swin, and (**c**) DeiT.

To further investigate the classification behavior of the individual transformer models, the corresponding confusion matrices are presented in Figure 7. The confusion matrices provide a detailed visualization of correctly and incorrectly classified gender samples, complementing the quantitative performance metrics reported in Table 6.

Consistent with the numerical results presented in Table 6, the ViT model produces the highest number of correctly classified samples while exhibiting the lowest number of false-positive and false-negative predictions among the individual transformer architectures. Swin Transformer also demonstrates strong classification capability, although with slightly higher misclassification rates than ViT. In contrast, DeiT exhibits comparatively greater confusion between the two gender classes, explaining its lower accuracy and F1-score. The confusion matrices therefore provide visual confirmation of the complementary classification characteristics of the evaluated transformer architectures.

A comparison of the regression metrics reveals that the performance difference between ViT and DeiT is relatively small, whereas Swin Transformer consistently provides lower prediction errors for age estimation. This observation suggests that hierarchical attention mechanisms are particularly suitable for modeling the gradual morphological changes associated with facial aging. Facial aging is characterized by localized texture variations, wrinkles, skin deformation, and structural changes occurring at multiple spatial scales. Consequently, the hierarchical feature representation learned by Swin Transformer enables more accurate estimation of continuous age values than architectures relying exclusively on global attention.

A similar trend is observed for gender classification. Although Swin Transformer achieves superior age estimation performance, ViT consistently outperforms the remaining architectures across all classification metrics. This finding indicates that the architectural requirements for regression and classification tasks are fundamentally different. While age estimation benefits from hierarchical multi-scale representations, gender classification relies more heavily on global semantic relationships captured through full-image self-attention. These complementary characteristics demonstrate that each transformer architecture specializes in different aspects of facial representation learning.

Overall, the experimental findings clearly indicate that no single transformer architecture simultaneously achieves the best performance for both age estimation and gender classification. Instead, each model exhibits task-specific strengths arising from its underlying attention mechanism and feature extraction strategy. The convergence behavior illustrated in Figure 6, together with the classification characteristics visualized in Figure 7, provides strong evidence that the three transformer architectures learn complementary facial representations. These observations establish a solid motivation for integrating the individual models through ensemble learning.

Therefore, rather than selecting a single best-performing transformer, the proposed framework combines the complementary capabilities of ViT, Swin Transformer, and DeiT using ensemble learning. The following section investigates whether integrating these heterogeneous transformer models through both standard averaging and the proposed TAWE strategy can further improve the robustness and predictive accuracy of simultaneous age estimation and gender classification [17].

### 3.2. Analysis of Ensemble Learning Performance

The complementary strengths observed among the individual transformer architectures motivated the development of an ensemble learning framework to further improve demographic prediction performance. Although each transformer model demonstrated competitive performance independently, the experimental results presented in the previous section revealed that no single architecture consistently achieved the best results for both age estimation and gender classification. Specifically, Swin Transformer produced the lowest regression errors for age estimation, whereas ViT achieved the highest classification performance for gender prediction. This complementary behavior suggests that integrating multiple transformer models could produce more robust and generalized predictions than relying on any individual architecture.

To investigate this hypothesis, two ensemble learning strategies were evaluated in this study: a conventional Standard Ensemble, in which the outputs of all transformer models were combined using equal weights, and the proposed TAWE, which assigns task-specific weights to each transformer according to its validation performance. The comparative experimental results are summarized in Table 7.

As presented in Table 7, both ensemble approaches improve age-estimation performance relative to the strongest individual MTL age-regression model, while their gender-classification performance remains comparable to that of the individual models. Even the Standard Ensemble improves the regression performance by reducing the MAE from 5.1496 (best individual model) to 4.8972, while simultaneously decreasing the RMSE from 6.7717 to 6.4960. Similar improvements are also observed for gender classification, where the Standard Ensemble achieves an accuracy of 94.37% and an F1-score of 94.70%, indicating that equal-weight averaging successfully reduces prediction variance among the individual transformer models [18].

The proposed method achieves the lowest age estimation errors with an MAE of 4.8905 and an RMSE of 6.4674, while simultaneously obtaining the highest gender classification performance with an accuracy of 94.86%, precision of 95.25%, recall of 95.08%, and an F1-score of 95.17%. The proposed TAWE achieved the best point estimates among the evaluated ensemble strategies, obtaining the lowest reported age-estimation errors and the highest reported gender-classification metrics. However, the improvement in age estimation over the standard equal-weight ensemble was modest and not statistically significant. These results demonstrate that assigning task-dependent importance to individual transformer architectures enables the ensemble to exploit their complementary representation capabilities more effectively than conventional equal-weight averaging.

The improvement achieved by the proposed weighted ensemble can be explained by the heterogeneous learning characteristics of the transformer architectures. Swin Transformer, owing to its hierarchical shifted-window attention mechanism, captures localized facial texture changes and age-related morphological patterns more effectively, thereby contributing more strongly to age estimation. Conversely, the global self-attention mechanism employed by ViT provides highly discriminative representations for gender classification by modeling long-range facial relationships across the entire image. Rather than forcing identical contributions from all models, the proposed ensemble emphasizes the transformer architecture that is more suitable for each prediction task. Consequently, the ensemble benefits simultaneously from both local and global feature representations.

Another important observation is that the proposed weighted ensemble provides slightly improved aggregate classification performance compared with the standard equal-weight ensemble. By combining predictions from heterogeneous transformer architectures, the ensemble exploits complementary information across the evaluated models. This complementary decision fusion may reduce the influence of model-specific prediction errors, although the present experiments do not directly quantify model stability or demographic bias [19].

**Figure 8:**
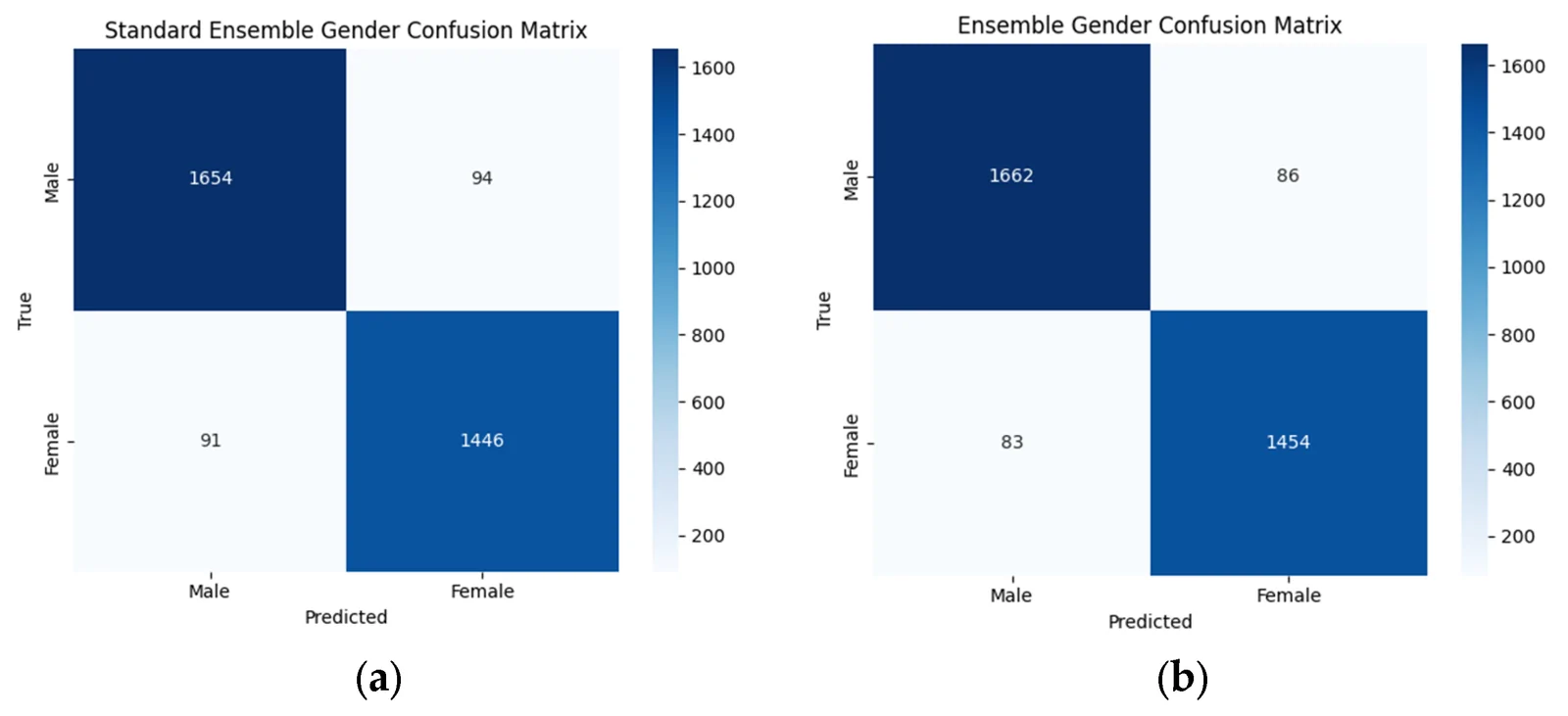
Confusion matrices of the evaluated ensemble learning strategies for gender classification: (**a**) Standard Ensemble and (**b**) Proposed TAWE. The confusion matrices illustrate the corresponding changes in correctly and incorrectly classified samples between the standard equal-weight ensemble and TAWE.

The confusion matrices presented in Figure 8 provide additional insight into the classification performance of the evaluated ensemble strategies. Compared with the Standard Ensemble, the proposed TAWE yields a higher number of correctly classified samples while simultaneously reducing both false-positive and false-negative predictions. These visual observations are fully consistent with the quantitative improvements reported in Table 7 and further confirm the effectiveness of the proposed weighting strategy for multi-task facial demographic analysis.

Overall, the experimental results demonstrate that ensemble learning substantially enhances the predictive capability of transformer-based MTL models. More importantly, the proposed task-aware weighting strategy provides task-specific model contributions based on validation performance and achieves the best point estimates among the evaluated ensemble strategies, while the improvement in age estimation remains modest. This task-specific validation-based weighting strategy enables the proposed framework to effectively exploit the complementary strengths of the transformer backbones across continuous age estimation and binary gender classification, resulting in the best point estimates among the evaluated ensemble strategies while maintaining comparable age-estimation performance.

### 3.3. Comparative Performance Analysis

To provide contextual information regarding the performance of the proposed framework, a comparative analysis was conducted with representative studies reported on the UTKFace dataset. The selected studies include conventional CNN-based approaches as well as more recent deep learning architectures addressing facial age estimation and gender classification [20]. The primary objective of this comparison is to provide contextual information on the proposed transformer-based MTL framework relative to previously published methods, while considering differences in task formulations and evaluation protocols. The comparative results are summarized in Table 8.

As shown in Table 8, the representative studies address facial demographic analysis using different problem formulations and evaluation strategies. Some studies focus on age-group classification rather than continuous age estimation, whereas others address only age prediction without considering gender classification. Accordingly, Table 8 should not be interpreted as a direct state-of-the-art ranking, and no claim of superiority over the reported studies is made. Consequently, numerical comparisons across these studies should be interpreted with caution because the learning objectives, dataset partitions, preprocessing procedures, and evaluation protocols may differ across studies.

The CNN-based model proposed by Dey et al. [21] reported high gender classification accuracy on the UTKFace dataset while simultaneously performing age-group classification. Similarly, the FINet/INFINet framework developed by Akgül [22] addressed age-group and gender classification within a unified architecture. Although these studies reported high gender prediction performance, age estimation was formulated as a classification problem rather than a continuous regression task. Therefore, their reported age-group classification accuracies are not directly comparable with regression-based approaches that estimate continuous age values.

Among the regression-based methods, Tanveer et al. [23] reported an MAE of 4.37 using a comparative evaluation of CNNs and ViTs. Likewise, Kocoń and Pawlukiewicz [24] achieved an MAE of 3.82 within a unified CNN-based framework while also reporting a gender classification accuracy of 93.50%. These results indicate that specialized architectures optimized primarily for age regression have reported lower age estimation errors on the UTKFace dataset.

Although the proposed framework does not report the lowest MAE among the studies summarized in Table 8, it addresses multiple prediction tasks within a unified architecture. First, the proposed approach simultaneously performs continuous age estimation and gender classification within a unified MTL architecture. Unlike methods that focus exclusively on a single prediction task, the proposed framework learns shared facial representations for simultaneous continuous age estimation and gender classification. This shared learning formulation enables the same backbone to support both prediction tasks within a unified model, potentially allowing information learned for one task to contribute to the other.

Second, the proposed framework is built entirely upon modern Transformer architectures, including ViT, Swin Transformer, and DeiT. These architectures employ different attention mechanisms and representational structures that can capture global contextual relationships and hierarchical visual patterns. By integrating these complementary Transformer backbones through the proposed TAWE, the framework combines representations derived from different architectural characteristics for the two demographic prediction tasks.

The quantitative results further characterize the performance of the proposed approach. The proposed framework achieves an MAE of 4.8905 and an RMSE of 6.4674 for age estimation while simultaneously obtaining 94.86% accuracy and 95.17% F1-score for gender classification. These results indicate that the proposed framework provides competitive age estimation and gender classification performance within a unified multi-task architecture. From a methodological perspective, simultaneous prediction of age and gender provides a unified framework for applications requiring multiple facial analysis outputs, although the suitability of such systems for specific real-world applications depends on application-specific validation, privacy requirements, and fairness considerations.

Another observation from Table 8 is that several of the summarized studies rely on a single backbone architecture. In contrast, the proposed framework combines multiple Transformer backbones through ensemble learning, allowing their task-specific contributions to be weighted according to validation performance. The experimental results presented in Section 3.2 indicate that the proposed task-specific validation-based weighting strategy assigns different contributions to ViT, Swin Transformer, and DeiT according to their observed task-specific performance. This formulation allows the ensemble to exploit complementary characteristics of the individual backbones, although the magnitude of the improvement varies across tasks and evaluation metrics. This observation supports the use of task-specific ensemble weighting when heterogeneous architectures exhibit different strengths across facial analysis tasks.

Overall, the comparative analysis indicates that the proposed framework provides a unified combination of continuous age estimation and gender classification while incorporating MTL and Transformer-based ensemble modeling. Although certain specialized models report lower age estimation errors, the proposed framework jointly addresses continuous age estimation and gender classification within a single multi-task ensemble framework. These findings suggest that combining MTL with task-specific Transformer ensemble strategies is a feasible approach for jointly addressing facial age estimation and gender classification within the evaluated UTKFace setting. However, further evaluation on independent datasets is required to determine the generalizability of the proposed framework to different populations and acquisition conditions.

The results reported in Section 3.1, Section 3.2 and Section 3.3 collectively indicate that the evaluated Transformer architectures exhibit different task-specific performance characteristics and that combining their predictions through task-specific validation-based weighting can provide complementary benefits across the evaluated tasks and metrics. To further investigate the contribution of the individual architectural components and quantify the effect of the proposed ensemble strategy, an ablation analysis is presented in the following section.

### 3.4. Ablation Study

To better understand the contribution of each component of the proposed framework, an ablation study was conducted using the same experimental settings described in Section 2. Rather than introducing additional architectures, the analysis progressively examines the performance obtained by the individual transformer models, the standard ensemble strategy, and the proposed TAWE. This evaluation aims to quantify the effectiveness of ensemble learning and to determine whether assigning task-specific weights provides measurable improvements over conventional equal-weight averaging. The experimental results of the ablation analysis are summarized in Table 9.

As shown in Table 9, the three transformer architectures exhibit complementary prediction characteristics. Swin Transformer provides the lowest regression errors for age estimation, indicating that its hierarchical shifted-window attention mechanism effectively captures localized age-related facial features across multiple spatial scales. In contrast, ViT achieves the highest gender classification accuracy among the individual models, demonstrating the effectiveness of global self-attention in learning holistic facial representations that are highly discriminative for gender recognition. Although DeiT produces comparatively lower predictive performance, it contributes additional diversity that becomes beneficial when integrated within an ensemble framework.

The first ablation stage evaluates the impact of conventional ensemble learning. Compared with the best-performing individual model, the Standard Ensemble decreases the MAE from 5.1496 to 4.8972 while simultaneously reducing the RMSE from 6.7717 to 6.4960. Although the classification accuracy (94.37%) is slightly lower than that of ViT (94.58%), the ensemble achieves a more balanced performance across both regression and classification tasks. These findings indicate that averaging predictions from heterogeneous transformer architectures effectively suppresses model-specific prediction errors and reduces the influence of individual model prediction errors and improves aggregate regression performance.

The second ablation stage investigates the contribution of the proposed TAWE. Instead of assigning identical contributions to all transformer models, the proposed strategy determines independent weights for each task using validation performance. Consequently, Swin Transformer contributes more strongly to age estimation, whereas ViT receives a larger contribution during gender prediction. This task-specific validation-based weighting strategy enables the framework to exploit the task-specific strengths of each architecture while reducing the influence of comparatively weaker predictions.

The effectiveness of this weighting strategy is clearly demonstrated by the quantitative results presented in Table 9. The proposed TAWE achieves the lowest regression errors, reducing the MAE to 4.8905 and the RMSE to 6.4674. Simultaneously, it produces the highest gender classification performance with an accuracy of 94.86% and an F1-score of 95.17%. Although the numerical improvements over the Standard Ensemble are modest, TAWE achieves lower MAE and RMSE and higher gender classification accuracy and F1-score than the Standard Ensemble. However, the age-estimation improvement is not statistically significant, whereas the improvement in gender classification accuracy is statistically significant. These results indicate that task-specific validation-based weighting can provide a statistically supported improvement in gender classification while maintaining comparable age-estimation performance.

An important observation derived from the ablation study is that the performance improvement cannot be attributed solely to increasing the number of models within the ensemble. Instead, the improvement originates from assigning appropriate responsibilities to each transformer according to its learning characteristics. This observation demonstrates that ensemble diversity alone is insufficient for maximizing predictive performance; rather, effective integration requires exploiting the complementary strengths of heterogeneous architectures in a task-dependent manner.

Overall, the ablation analysis provides evidence that the shared MTL architecture, heterogeneous transformer backbones, and task-specific ensemble fusion contribute to the overall behavior of the proposed framework, while individual components may provide different benefits across the evaluated metrics. The shared MTL architecture enables efficient representation learning for both demographic attributes, the transformer backbones provide complementary feature extraction capabilities, and the proposed TAWE effectively combines these heterogeneous representations into a unified prediction framework. These findings validate the design philosophy of the proposed architecture and demonstrate that task-specific validation-based ensemble learning constitutes an important component of the proposed ensemble design.

Finally, the proposed weighted ensemble introduces additional computational overhead compared with individual transformer models. The computational characteristics of the proposed framework are therefore examined separately in Section 3.5.

#### 3.4.1. Comparison with Single-Task Learning

To further investigate the contribution of the proposed MTL framework, additional experiments were conducted using independently trained single-task transformer models. In these experiments, ViT, Swin Transformer, and DeiT were separately optimized for age estimation and gender classification, without sharing feature representations between the two tasks. All single-task models were trained under the same experimental protocol as the proposed framework, including identical data partitions, preprocessing procedures, input resolution, optimization settings, and evaluation metrics. This design enables a fair comparison between independent task learning and the proposed shared MTL architecture.

The quantitative comparison between the independently trained single-task models and the proposed TAWE is presented in Table 10.

As shown in Table 10, the proposed TAWE framework achieved the lowest MAE (4.8905) and RMSE (6.4674), outperforming all independently trained single-task transformer models in age estimation. Compared with the strongest single-task baseline (Swin Transformer), TAWE reduced the MAE from 5.0231 to 4.8905 and the RMSE from 6.7534 to 6.4674, demonstrating that combining complementary transformer representations within a shared multi-task framework improves regression performance.

For gender classification, the independently trained ViT model achieved a higher classification accuracy (95.53%) than the proposed TAWE framework (94.86%), whereas TAWE achieved a marginally higher F1-score (95.17%) than the single-task ViT model (95.16%). This observation suggests that optimizing a transformer exclusively for a single classification objective can provide a marginal advantage in terms of classification accuracy, while task-aware ensemble fusion can achieve comparable classification performance when evaluated using the F1-score. However, such single-task optimization is focused exclusively on gender classification and does not provide the unified age estimation and gender classification capability offered by the proposed multi-task framework.

Overall, these results indicate that the primary advantage of the proposed framework is to provide a unified multi-task solution capable of simultaneously performing age estimation and gender classification. By combining multi-task representation learning with task-aware ensemble fusion, TAWE exploits the complementary strengths of the transformer backbones and achieves the best point estimates among the evaluated ensemble strategies, while maintaining competitive gender classification performance. These results support the effectiveness of the proposed framework within the evaluated UTKFace setting, while further validation on independent datasets is needed to assess its generalizability to broader real-world applications.

#### 3.4.2. Statistical Significance Analysis

To further examine whether the performance improvements achieved by the proposed TAWE are statistically meaningful rather than arising from random variation, statistical significance analyses were performed using the predictions obtained from the identical 3285 test samples. Since age estimation is formulated as a regression problem, the sample-wise absolute prediction errors of the standard ensemble and the proposed TAWE were compared using the non-parametric Wilcoxon signed-rank test. For gender classification, where both models produced paired binary predictions on the same test samples, the Exact McNemar test was employed to assess whether the observed difference in classification accuracy was statistically significant. The results of the statistical analyses are summarized in Table 11.

As shown in Table 11, although the proposed TAWE achieved a lower MAE than the conventional equal-weight ensemble (4.8905 vs. 4.8972), the Wilcoxon signed-rank test indicates that this improvement is not statistically significant (*p* = 0.5816). Considering that the absolute difference between the two MAE values is relatively small, this outcome suggests that both ensemble strategies exhibit highly similar regression behavior on the UTKFace test set. Therefore, the proposed task-aware weighting strategy preserves the age estimation capability of the standard ensemble while providing a modest numerical improvement.

In contrast, the Exact McNemar test demonstrates that the improvement in gender classification is statistically significant (*p* = 0.0166). This finding indicates that the task-specific weighting mechanism successfully exploits the complementary strengths of the individual transformer backbones, leading to a meaningful reduction in gender misclassification errors. Consequently, although the regression improvement remains numerically small and statistically non-significant, the proposed TAWE provides statistically significant benefits in gender classification accuracy while maintaining comparable age-estimation performance.

Overall, these statistical analyses provide additional evidence regarding the reliability of the observed performance differences. In particular, the statistically significant improvement in gender classification accuracy provides supporting evidence for the effectiveness of the task-specific validation-based weighting strategy, whereas the age-estimation improvement should be interpreted as a modest numerical gain rather than a statistically significant improvement.

#### 3.4.3. Effect of Task-Aware Weighting

The effectiveness of the proposed TAWE was further investigated by analyzing the task-specific weighting strategy adopted for age estimation and gender classification. Unlike conventional ensemble methods that assign identical contributions to all constituent models, the proposed framework allocates different model weights according to their validation performance for each prediction task. This design enables the ensemble to exploit the complementary strengths of different transformer architectures while maintaining a simple and interpretable fusion mechanism.

The task-specific weights determined during the validation stage are summarized in Table 12. As can be observed, different transformer models receive different levels of contribution depending on the prediction objective. Swin Transformer was assigned the largest weight for age estimation (0.55), whereas ViT received the highest contribution for gender classification (0.55). DeiT retained a smaller yet non-zero contribution in both tasks, providing complementary predictive information to the ensemble.

The selected weights closely reflect the behavior of the individual transformer models reported in Section 3.1. Swin Transformer achieved the lowest MAE and RMSE values, indicating superior capability for modeling continuous facial aging patterns. Consequently, assigning the largest contribution to Swin Transformer for age estimation enables the ensemble to preserve its strong regression performance. Conversely, ViT achieved the highest gender classification accuracy and F1-score among the individual transformer models, motivating its larger contribution to the gender prediction branch. Rather than assuming that a single transformer architecture is equally suitable for all demographic prediction tasks, TAWE explicitly exploits these task-dependent performance differences.

This task-aware weighting strategy differs fundamentally from conventional equal-weight averaging, which assumes identical predictive capability across all constituent models. Instead of enforcing equal contributions, TAWE reflects the empirical strengths observed during validation through fixed task-specific weights, while avoiding additional trainable parameters or complex optimization procedures. Consequently, the proposed fusion strategy preserves the transparency and computational efficiency of classical ensemble learning while enabling task-specific decision fusion tailored to each prediction task.

The effectiveness of this strategy is further supported by the quantitative results presented in Table 7, where the proposed TAWE achieved the best point estimates among the evaluated ensemble strategies, with the lowest reported age-estimation errors and the highest reported gender-classification metrics; however, the improvement in age estimation over the standard equal-weight ensemble was modest and not statistically significant. Although the improvement in age estimation is numerically modest, the task-specific validation-based weighting strategy preserved the strong regression capability of the ensemble while improving gender classification accuracy. Moreover, as demonstrated by the statistical significance analysis presented in Section 3.4.2, the improvement in gender classification was statistically significant according to the Exact McNemar test (*p* = 0.0166), whereas the difference in age estimation was not statistically significant. These findings suggest that the proposed task-specific weighting mechanism primarily provides a statistically supported improvement in gender classification accuracy while maintaining competitive age-estimation performance.

Overall, the experimental results suggest that the performance differences observed with TAWE are related not only to combining multiple transformer models but also to assigning task-specific contributions according to the empirical strengths of each architecture. This observation indicates that validation-based task-specific weighting provides a simple and computationally lightweight fusion strategy alternative to more sophisticated fusion strategies, while preserving model interpretability and avoiding the increased optimization complexity associated with learnable fusion networks.

#### 3.4.4. Demographic Subgroup Analysis

To further investigate the behavior of the proposed TAWE framework across demographic subgroups, a post hoc subgroup analysis was performed using the fixed independent UTKFace test set comprising 3285 samples. The analysis was conducted using the final TAWE predictions, and no additional training, model selection, or test-set tuning was performed. Age estimation was evaluated using Mean Absolute Error (MAE) and Root Mean Squared Error (RMSE), whereas gender classification was evaluated using Accuracy and Macro-F1. Macro-F1 was used for the subgroup analysis to provide a class-balanced measure across the two dataset gender labels and to avoid ambiguity associated with the selection of the positive class.

For the main gender-classification evaluation, F1-score was calculated for the dataset-defined positive class. For the demographic subgroup analysis, Macro-F1 was additionally reported to provide a class-balanced measure across the two gender labels and to avoid dependence on the choice of positive class.

As shown in Table 13, the age-stratified results indicate that gender classification performance remains relatively stable across the evaluated age intervals. Gender classification Accuracy ranges from 94.25% to 95.14%, while Macro-F1 ranges from 93.04% to 95.04%. In contrast, age-estimation error varies more substantially across age groups. The lowest MAE is obtained for the 21–30 age group (3.9919 years), whereas the highest MAE is observed for the 51–60 group (7.4988 years). A similar pattern is observed for RMSE, which increases from 5.3426 years in the 21–30 group to 9.6405 years in the 51–60 group. These results indicate that the classification component maintains relatively stable performance across the evaluated age intervals, whereas continuous age estimation becomes more challenging in the older age groups.

The results in Table 14 show that TAWE maintains relatively consistent gender classification performance across the five ethnicity-code groups represented in the independent test set. Gender classification Accuracy ranges from 93.17% to 95.80%, while Macro-F1 ranges from 93.17% to 95.70%. Age MAE ranges from 3.8335 to 5.1853 years, indicating some variation in regression performance across the groups. Ethnicity code 2 has the lowest age-estimation error, whereas ethnicity code 3 has the highest gender classification Accuracy and Macro-F1. However, the subgroup sizes are unequal, particularly for ethnicity code 4 (*N* = 205). Therefore, these differences are interpreted as descriptive subgroup variations rather than definitive evidence regarding demographic fairness or the absence of bias.

The gender-stratified results in Table 15 show closely matched performance between the two dataset gender labels. Age MAE is 4.9442 years for male-labeled samples and 4.8295 years for female-labeled samples, corresponding to a difference of 0.1147 years. Gender classification accuracy is 95.08% and 94.60%, respectively. The results therefore indicate relatively small descriptive differences between the two binary gender-label groups represented in UTKFace. These findings should nevertheless be interpreted within the limitations of the dataset-defined binary labels and should not be considered evidence of fairness across broader gender identities or populations.

To verify consistency with the principal evaluation, the subgroup analyses were derived from the same 3285 independent test samples used for the main TAWE evaluation. Recalculation from all final TAWE predictions reproduced the overall age MAE of 4.8905 years, age RMSE of 6.4674 years, and gender classification Accuracy of 94.86%. The subgroup sample counts also sum to 3285 for each demographic grouping. These consistency checks confirm that the subgroup analysis was conducted on the same independent test set without modifying the trained models or prediction procedure.

### 3.5. Computational Complexity Analysis

In addition to predictive performance, computational efficiency is an important consideration for the practical deployment of transformer-based facial analysis systems. While Section 2 summarized the architectural characteristics of the evaluated transformer backbones in Table 3, this section quantitatively analyzes their computational requirements during inference. The comparison includes the number of trainable parameters, floating-point operations (FLOPs), model size, GPU memory consumption, and inference time, as summarized in Table 16.

As shown in Table 16, the three transformer backbones exhibit substantially different computational characteristics despite being trained under the same MTL framework. Swin-T is the most computationally efficient architecture, requiring only 28.3 million trainable parameters and 4.51 GFLOPs, which are considerably lower than those of both ViT-B/16 and DeiT-B. This lightweight design is mainly attributed to the shifted-window self-attention mechanism, which restricts self-attention computations to local windows while preserving hierarchical feature extraction. Consequently, Swin-T achieves the lowest computational complexity and memory consumption among the evaluated transformer architectures.

In contrast, both ViT-B/16 and DeiT-B employ global self-attention over fixed image patches, resulting in significantly higher computational requirements. Although DeiT-B adopts a more data-efficient training strategy through knowledge distillation, its inference complexity remains comparable to that of ViT because both architectures share nearly identical transformer backbones. Consequently, ViT-B/16 and DeiT-B require approximately 86.5 million parameters and around 17 GFLOPs, representing a substantially larger computational burden than Swin-T.

The proposed TAWE naturally introduces additional computational cost because predictions from all three transformer models must be generated before the final decision is obtained. As expected, the complete ensemble requires 201.43 million trainable parameters, 38.40 GFLOPs, approximately 761 MB of model storage, and 823 MB of GPU memory during inference. The average inference time also increases to 39.49 ms per image, reflecting the cumulative execution of the three transformer backbones.

Nevertheless, it is important to emphasize that the increased computational cost originates almost entirely from executing multiple backbone networks rather than from the proposed weighting mechanism itself. The TAWE fusion stage performs only simple weighted averaging for age estimation and weighted probability aggregation for gender classification, introducing negligible computational overhead without requiring additional trainable parameters or iterative optimization. Therefore, the computational complexity of the proposed framework is primarily determined by the underlying transformer models instead of the ensemble strategy itself.

The increased computational cost of the proposed TAWE framework should be considered together with its predictive performance characteristics. As demonstrated in Table 7, TAWE achieved the best point estimates among the evaluated ensemble strategies, with an age MAE of 4.8905, an RMSE of 6.4674, a gender classification accuracy of 94.86%, and an F1-score of 95.17%. Compared with the standard equal-weight ensemble, TAWE reduced the age MAE from 4.8972 to 4.8905 and the RMSE from 6.4960 to 6.4674, while increasing gender classification accuracy from 94.37% to 94.86%. However, the age-estimation differences were modest and the MAE improvement was not statistically significant, whereas the improvement in gender classification accuracy was statistically significant. These results indicate that the additional computational cost of TAWE is associated with task-specific ensemble prediction and a statistically supported improvement in gender classification accuracy, while the gains in age estimation remain limited. Since the fusion stage introduces no additional trainable parameters, the additional computational burden is primarily attributable to the simultaneous execution of the three transformer backbones.

The modular architecture of the proposed framework also provides flexibility in selecting an appropriate model configuration according to computational requirements. For applications with limited computational resources or strict latency constraints, an individual transformer model may be selected according to the target task. When simultaneous age estimation and gender classification are required and the available computational resources permit ensemble inference, the complete TAWE framework can be employed to combine the predictions of the three transformer backbones. Thus, model selection can be determined according to the computational constraints and prediction requirements of the intended application.

Overall, the computational analysis demonstrates that the proposed TAWE framework requires substantially greater computational resources than the individual transformer models because predictions from all three backbones must be generated during inference. The fusion stage itself requires no additional trainable parameters and introduces only negligible computational overhead. While TAWE achieved the best point estimates among the evaluated ensemble strategies, the corresponding improvement over equal-weight averaging was modest for age estimation and statistically significant for gender classification accuracy. Future research may further reduce the computational burden through model compression, knowledge distillation, pruning, quantization, or dynamic inference strategies while preserving the task-specific fusion capability of the proposed framework.

### 3.6. Discussion

The experimental results presented throughout this study indicate that transformer-based MTL combined with task-specific validation-based weighting provides a unified framework for simultaneous age estimation and gender classification. Unlike approaches that rely on a single backbone architecture or optimize each task independently, the proposed framework combines heterogeneous Transformer models within a unified multi-task prediction pipeline. The results obtained from the individual model evaluation, ensemble comparison, ablation studies, statistical significance analysis, and computational complexity assessment indicate that combining multiple transformer architectures through task-specific validation-based weighting can provide complementary predictive benefits while maintaining a unified multi-task framework.

One of the important findings of this study is that different Transformer architectures exhibit complementary task-specific representation characteristics. As demonstrated in Section 3.1 and Section 3.4, Swin Transformer achieved the lowest age estimation errors among the individual MTL models, whereas ViT provided the highest gender classification performance. These observations are consistent with the different representational characteristics of the evaluated architectures, with Swin Transformer employing hierarchical shifted-window attention and ViT relying on global self-attention. Rather than relying on a single backbone for both prediction tasks, the proposed framework exploits the observed task-specific differences through independent task-specific weighting. Accordingly, TAWE assigns larger fixed contributions to Swin Transformer for age estimation and to ViT for gender classification based on their validation performance. This task-specific formulation distinguishes the proposed ensemble from conventional equal-weight averaging by assigning different validation-based contributions to the prediction tasks [25].

The proposed TAWE should not be interpreted as introducing a fundamentally new arithmetic operation for ensemble learning, since weighted averaging is a well-established fusion mechanism. Instead, its contribution lies in formulating a task-specific validation-based weighting strategy within a unified multi-transformer MTL framework. The same heterogeneous Transformer models are therefore not required to contribute equally to both prediction tasks. The fixed weights are determined from validation performance and reflect the empirically observed complementary strengths of the individual architectures. In particular, the larger contribution assigned to Swin Transformer for age estimation and to ViT for gender classification provides a simple mechanism for exploiting task-specific differences without introducing additional trainable fusion parameters or complex optimization procedures. This formulation also provides a transparent alternative to more complex learnable fusion mechanisms such as stacking, gating, or optimized fusion.

The ablation studies further characterize the contribution of the proposed design choices. The comparison with independently trained single-task Transformer models indicates that the proposed multi-task framework provides a balanced solution for age estimation and gender classification, although individual single-task models may achieve higher performance on specific metrics. For gender classification, the independently trained ViT model achieved a higher classification accuracy (95.53%) than the proposed TAWE framework (94.86%), whereas TAWE achieved a marginally higher F1-score (95.17%) than the single-task ViT model (95.16%). This observation suggests that optimizing a Transformer exclusively for a single classification objective can provide a marginal advantage in terms of classification accuracy, while task-specific ensemble fusion can achieve comparable classification performance when evaluated using the F1-score. However, such single-task optimization is focused exclusively on gender classification and does not provide the unified age estimation and gender classification capability offered by the proposed multi-task framework. Thus, the primary advantage of the proposed framework is not necessarily to maximize every individual evaluation metric, but rather to jointly address two prediction tasks within a unified architecture.

The comparison between the equal-weight ensemble and TAWE also provides a more nuanced interpretation of the contribution of task-specific weighting. TAWE reduced the age MAE from 4.8972 to 4.8905 and the RMSE from 6.4960 to 6.4674, while increasing gender classification accuracy from 94.37% to 94.86%. Although the numerical improvement in age estimation is modest, statistical analysis showed that the difference in age MAE was not statistically significant (Wilcoxon signed-rank test, *p* = 0.5816). In contrast, the improvement in gender classification accuracy was statistically significant according to the Exact McNemar test (*p* = 0.0166). Therefore, the results should not be interpreted as evidence of statistically significant improvement across all prediction metrics. Instead, they indicate that the task-specific validation-based weighting strategy provides a statistically supported improvement in gender classification accuracy while maintaining comparable age-estimation performance. This distinction is important when interpreting the contribution of TAWE, particularly because the primary benefit of task-specific weighting is to exploit complementary model characteristics rather than to guarantee substantial improvements for every individual metric.

Another important observation concerns the computational characteristics of the proposed framework. As discussed in Section 3.5, integrating three Transformer backbones inevitably increases inference cost compared with individual models. The computational analysis showed that the additional burden originates primarily from executing multiple backbone networks rather than from the weighting strategy itself. Since TAWE introduces no additional trainable parameters, its fusion operation adds only limited computational overhead relative to the cost of the three backbone models. The complete TAWE framework has 201.43 million parameters, 38.40 GFLOPs, 822.99 MB GPU memory usage, and an average inference time of 39.49 ms, compared with substantially lower computational requirements for the individual Transformer models. Therefore, the proposed framework preserves the methodological simplicity and interpretability of conventional ensemble learning while providing task-specific fusion without introducing an additional trainable fusion network. Individual Transformer models may be considered in resource-constrained settings, whereas the complete TAWE framework requires additional computational resources because of the simultaneous execution of three backbone networks. The appropriate configuration should therefore depend on the computational requirements and risk profile of the intended application.

Several limitations should be acknowledged when interpreting these findings. First, all experiments were conducted exclusively on the UTKFace dataset. Although UTKFace contains variability in facial appearance, age, gender, and ethnicity, evaluation on additional independent datasets would be necessary to further assess the generalizability of the proposed framework under different demographic distributions, image acquisition conditions, and annotation characteristics. Datasets such as MORPH-II, FG-NET, APPA-REAL, or IMDB-WIKI may provide relevant settings for future cross-dataset evaluation. Accordingly, the results reported in this study should be interpreted within the evaluated UTKFace experimental setting rather than as evidence of generalization to all facial image populations. Although the subgroup analysis provides additional descriptive information regarding model performance across the demographic categories represented in UTKFace, it remains subject to the same dataset-specific limitations. In particular, the subgroup sizes are unequal, and the available demographic labels reflect the annotation structure of UTKFace. Therefore, the observed subgroup performance differences should not be interpreted as definitive evidence of demographic fairness or generalizable performance parity. Future studies should extend the subgroup analysis to larger and independent datasets with more diverse demographic distributions and incorporate formal statistical analyses of subgroup disparities.

The present study follows the binary gender annotations provided by the UTKFace dataset and therefore performs gender prediction strictly according to the available dataset labels. Consequently, the proposed framework should not be interpreted as inferring an individual’s gender identity or broader social constructs of gender. Instead, the classification task is limited to the binary gender labels defined within the benchmark dataset. The subgroup analysis presented in Section 3.4.4 provides an initial descriptive assessment of performance across these dataset-defined gender labels; however, it does not establish fairness across broader gender identities or populations. Future research should therefore investigate demographic bias, subgroup-specific performance, and fairness-aware learning strategies, while also considering more inclusive approaches to demographic analysis.

The additional subgroup analysis provides further insight into the distribution of TAWE performance across the demographic categories represented in the independent test set. The age-stratified results show relatively stable gender classification performance across the evaluated age intervals, with Accuracy ranging from 94.25% to 95.14% and Macro-F1 ranging from 93.04% to 95.04%. In contrast, age-estimation errors increase across the older age intervals, with MAE increasing from 3.9919 years in the 21–30 group to 7.4988 years in the 51–60 group. The ethnicity-code analysis also shows generally consistent gender classification performance, although variation in age-estimation error is observed across the represented groups. Similarly, the gender-stratified analysis indicates closely matched performance between the two dataset gender labels. These findings provide a descriptive assessment of subgroup performance within the evaluated UTKFace test set. However, the unequal subgroup sizes and the use of a single dataset limit the extent to which these results can be interpreted as evidence of demographic fairness or generalizable performance parity. Therefore, larger and independent datasets with diverse demographic distributions, together with formal statistical fairness analyses, are needed to further investigate potential subgroup disparities.

#### Ethical, Privacy, and Fairness Considerations

Facial demographic prediction raises important ethical, privacy, and fairness considerations. Demographic composition, image acquisition conditions, and annotation practices within a dataset may introduce systematic biases that can affect model performance across different population groups. The subgroup analysis presented in Section 3.4.4 provides a descriptive assessment of TAWE performance across age intervals, ethnicity-code groups, and the binary gender labels represented in the UTKFace test set. However, subgroup-level performance consistency should not be interpreted as establishing demographic fairness or the absence of bias, particularly given the unequal subgroup sizes and the use of a single dataset. More comprehensive fairness assessment requires evaluation on larger and independent datasets with diverse demographic distributions, together with formal statistical analysis of subgroup disparities.

Facial images and inferred demographic attributes may constitute sensitive personal information depending on the application and applicable regulations. Therefore, practical use of such systems should incorporate appropriate privacy protection, data governance, and informed-consent requirements where applicable. The reported experimental performance should not be interpreted as evidence of suitability for surveillance applications, biometric decision-making, or other high-stakes applications. Before deployment in sensitive contexts, additional validation, subgroup-specific fairness assessment, privacy safeguards, and application-specific risk analysis would be required. These considerations are particularly important because aggregate predictive performance alone does not establish the ethical or regulatory suitability of a facial demographic prediction system.

An additional direction for future research concerns the exploration of alternative ensemble fusion mechanisms and multi-task loss weighting strategies. The present study adopts a fixed validation-based weighting strategy because of its transparency, interpretability, and computational efficiency. Although experiments involving different task-loss weight combinations and additional fusion strategies are beyond the scope of the present study, these approaches represent relevant directions for future investigation. In particular, stacking, learnable gating networks, uncertainty-aware weighting, mixture-of-experts architectures, and optimized task-loss weighting could be evaluated to determine whether they provide additional benefits under comparable experimental conditions. Such studies would also allow a more systematic assessment of the trade-offs among predictive performance, computational cost, interpretability, and optimization complexity.

Beyond alternative fusion strategies, several additional extensions may further improve the practical applicability of the proposed framework. Lightweight Transformer architectures, model compression, pruning, quantization, and knowledge distillation could be investigated to reduce computational requirements while assessing their effects on prediction performance. Similarly, self-supervised or contrastive representation learning could be investigated to improve representation learning under limited annotated data. Extending the proposed multi-task framework to additional facial analysis tasks, such as facial expression recognition, head pose estimation, or selected facial attribute analysis, may provide opportunities to investigate broader multi-task Transformer representations. Moreover, integrating Explainable Artificial Intelligence (XAI) techniques, including attention visualization and gradient-based attribution methods, may improve the transparency and interpretability of Transformer-based predictions and facilitate the analysis of model behavior [26].

Overall, the findings of this study indicate that combining complementary Transformer representations through MTL and task-specific validation-based weighting provides a unified approach for simultaneous facial age estimation and gender classification within the evaluated UTKFace setting. Rather than relying on a single Transformer architecture, the proposed framework combines heterogeneous representations with a fixed task-specific fusion mechanism that requires no additional trainable parameters and maintains methodological simplicity and interpretability. The proposed framework achieved the best point estimates among the evaluated ensemble strategies, while the statistical analysis indicates that the improvement over equal-weight averaging is statistically supported for gender classification accuracy but not for age estimation. These findings suggest that task-specific multi-Transformer learning provides a potential direction for future multi-task computer vision research, while further evaluation on independent datasets and across demographic subgroups is needed to assess generalizability, fairness, and suitability for broader real-world applications.

### 3.7. Qualitative Analysis

To complement the quantitative evaluation presented in the previous sections, representative qualitative prediction examples obtained from the proposed TAWE are summarized in Table 17. The selected samples include both successful predictions and representative failure cases from the UTKFace test set. For each facial image, the Ground-Truth (GT) and Predicted (Pred.) age and gender labels are reported together with the absolute age estimation error. This qualitative evaluation provides additional insight into the prediction behavior of the proposed framework beyond the aggregate performance metrics presented in the previous sections.

Overall, the qualitative results complement the quantitative findings reported in the preceding sections. The selected examples include several successful predictions, while the representative failure cases illustrate that prediction errors remain possible, particularly in age estimation.

The representative failure cases presented in Table 17 further illustrate the remaining challenges associated with facial demographic analysis. Although gender classification remains highly reliable for most samples, larger deviations occasionally occur in age estimation because facial aging is a continuous biological process characterized by considerable inter-subject variability. Consequently, individuals with similar chronological ages may exhibit noticeably different facial appearances, while subjects from different age groups may share similar facial characteristics. These findings are consistent with the regression results discussed throughout the previous sections.

Overall, the qualitative evaluation supports the quantitative performance analysis by visually demonstrating both the strengths and the remaining limitations of the proposed framework. While the selected examples include several successful predictions, the representative failure cases indicate that prediction errors remain possible, particularly in age estimation.

## 4. Conclusions

This study proposed a transformer-based MTL framework for the simultaneous estimation of age and classification of gender from facial images. Unlike conventional approaches that address these tasks independently, the proposed framework exploits shared facial representations while preserving task-specific prediction branches. To further benefit from the complementary characteristics of different transformer architectures, ViT, Swin Transformer, and DeiT were integrated through a TAWE, in which model contributions were determined independently for each prediction task according to their validation performance.

Experimental evaluation on the UTKFace dataset demonstrated that the three transformer backbones exhibit complementary strengths. Swin Transformer achieved the best age estimation performance, whereas ViT consistently produced the highest gender classification accuracy among the individual models. By combining these complementary capabilities through task-specific validation-based weighting, the proposed TAWE achieved the best point estimates among the evaluated ensemble strategies, obtaining an MAE of 4.8905, an RMSE of 6.4674, a gender classification accuracy of 94.86%, and an F1-score of 95.17%. Additional ablation studies, statistical significance analysis, computational complexity evaluation, and qualitative prediction examples provided further evidence regarding the effectiveness of the proposed framework within the evaluated UTKFace setting.

Beyond the quantitative improvements, the proposed framework offers a flexible and modular architecture that can be adapted to different deployment scenarios. Individual transformer models may be employed in resource-constrained environments, whereas the complete TAWE framework can be considered when simultaneous multi-task prediction and ensemble-based fusion are required and the available computational resources permit the additional inference cost. This modularity allows the model configuration to be selected according to computational requirements and prediction objectives. However, potential deployment in biometric, demographic, or other sensitive applications would require additional validation, fairness assessment, privacy safeguards, and application-specific risk analysis.

Despite these promising results, several limitations remain. Because all experiments were conducted on UTKFace, the present results should not be interpreted as evidence of cross-dataset generalization. Differences in demographic composition, image acquisition conditions, annotation practices, and dataset-specific biases may affect performance on other benchmarks. Moreover, although the proposed validation-based weighting strategy proved effective and computationally efficient, more sophisticated task-specific validation-based fusion approaches and alternative task-loss weighting strategies deserve further investigation. Future research will therefore focus on cross-dataset evaluation, lightweight transformer architectures, advanced task-specific validation-based fusion mechanisms, fairness-aware learning, XAI, and extending the proposed MTL framework to additional facial analysis tasks.

A post hoc demographic subgroup analysis further examined TAWE performance across age intervals, ethnicity-code groups, and the binary gender labels represented in the UTKFace test set. The analysis showed relatively stable gender classification performance across the evaluated subgroups, while age-estimation errors were higher in the older age intervals. These findings provide a descriptive assessment of subgroup behavior but do not establish demographic fairness or generalizable performance parity.

Overall, the results indicate that integrating complementary transformer architectures through a task-specific validation-based ensemble provides an effective and interpretable approach for simultaneous facial age estimation and gender classification. The proposed framework provides a basis for future multi-task transformer-based facial analysis research and highlights the potential of task-specific model weighting for exploiting complementary representations across prediction tasks.

## Figures and Tables

**Table 1:** Critical comparison between representative transformer-based facial demographic analysis studies and the proposed framework. Note: ✓ indicates that the corresponding characteristic is present or applicable, wherea ✗ indicates that it is absent or not applicable.

Study	Multi-Task Learning	Transformer	Multiple Backbones	Task-Specific Ensemble	Age Regression	Gender Classification
SwinFace [2]	✓	✓	✗	✗	✓	Facial
Liao et al. [3]	✓	✗	✗	✗	✓	✓
Han et al. [4]	✓	✗	✗	✗	✓	✓
HyperFace [5]	✓	✗	✗	✗	✗	✓
Proposed	✓	✓	✓	✓	✓	✓

**Table 2:** Distribution of the UTKFace dataset used in this study.

Dataset Split	Number of Images	Purpose
Training	10,385	Model learning
Validation	1833	Hyperparameter optimization and ensemble weight selection
Test	3285	Independent performance evaluation
Total	15,503	-

**Table 3:** Architectural specifications of the transformer backbones employed in this study.

Model	Input Size	Patch Size	Embedding Dimension	Parameters (M)	Main Characteristic
ViT-B/16	224 × 224	16 × 16	768	86.6	Global self-attention over fixed image patches
Swin-T	224 × 224	4 × 4	768	28.3	Hierarchical shifted-window self-attention
DeiT-B	224 × 224	16 × 16	768	86.5	Data-efficient transformer with knowledge distillation

**Table 4:** Conceptual comparison of representative ensemble fusion strategies and the proposed TAWE.

Fusion Strategy	Additional Trainable Parameters	Complexity	Interpretability	Used in This Study
Equal-weight averaging	No	Low	High	Yes
Validation-based weighting (TAWE)	No	Low	High	Yes
Stacking	Yes	Medium	Medium	No
Learnable gating	Yes	High	Low	No
Dynamic fusion	Yes	High	Low	No
Uncertainty weighting	Yes	Medium	Medium	No

**Table 5:** Experimental hyperparameters and computational environment used throughout the study.

Hyperparameter	Value
Deep learning framework	PyTorch 2.2.2
Programming language	Python 3.11
Operating system	Windows 11 Pro (64-bit)
CUDA Toolkit	CUDA 12.1
GPU	NVIDIA GeForce RTX 4060 (8 GB GDDR6)
CPU	Intel Core i7 (4.70 GHz)
RAM	32 GB DDR4
Pretrained weights	ImageNet
Input image size	224 × 224
Optimizer	AdamW
Initial learning rate	1 × 10^−4^
Weight decay	1 × 10^−4^
Batch size	32
Number of epochs	30
Random seed	42
Scheduler	Cosine Annealing LR
Early stopping	Based on validation loss
Loss function	Joint multi-task loss
Evaluation protocol	Independent test set

**Table 6:** Performance comparison of the individual transformer-based MTL models.

Model	MAE	RMSE	Acc (%)	Pre (%)	Rec (%)	F1-Score (%)
ViT	5.3908	7.1256	94.58	95.17	94.62	94.89
SwinTransformer	5.1496	6.7717	93.12	93.69	93.36	93.52
DeiT	5.3807	7.0764	91.75	92.56	91.88	92.22

**Table 7:** Performance comparison of the ensemble learning strategies.

Model	MAE	RMSE	Acc (%)	Pre (%)	Rec (%)	F1-Score (%)
Standard Ensemble	4.8972	6.4960	94.37	94.79	94.62	94.70
Weighted Ensemble (TAWE)	4.8905	6.4674	94.86	95.25	95.08	95.17

**Table 8:** Comparative performance analysis of the proposed framework and representative studies reported on the UTKFace dataset.

Study	Dataset	Method/Backbone	Age Estimation	Gender Classification	Task
Dey et al. [21]	UTKFace	CNN-based deep learning model	81.96% age classification accuracy	96.32% Acc	Age-group & gender classification
Akgül [22]	UTKFace	FINet/INFINet	72.00% age classification accuracy	90.50% Acc	Age-group & gender classification
Tanveer et al. [23]	UTKFace	CNN and ViT comparison	4.37 MAE	–	Age estimation
Kocoń and Pawlukiewicz [24]	UTKFace	Unified CNN-based multi-task network	3.82 MAE	93.50% Acc	Age regression & gender classification
Our study	UTKFace	Multi-task ViT + Swin Transformer + DeiT + Task-Aware Weighted Ensemble (TAWE)	4.8905 MAE/6.4674 RMSE	94.86% Acc/95.17% F1	Age regression & gender classification

**Table 9:** Ablation analysis of the proposed transformer-based MTL framework.

Configuration	MAE	RMSE	Acc (%)	F1-Score (%)
ViT	5.3908	7.1256	94.58	94.89
Swin Transformer	5.1496	6.7717	93.12	93.52
DeiT	5.3807	7.0764	91.75	92.22
Standard Ensemble	4.8972	6.4960	94.37	94.70
Proposed model (TAWE)	4.8905	6.4674	94.86	95.17

**Table 10:** Comparison between independently trained single-task models and the proposed TAWE framework.

Model	Age MAE	Age RMSE	Gender Accuracy	Gender F1
ViT—Single-Task	5.4762	7.1565	95.53%	95.16%
Swin—Single-Task	5.0231	6.7534	94.70%	94.31%
DeiT—Single-Task	5.4383	7.1862	94.64%	94.30%
Proposed (TAWE)	4.8905	6.4674	94.86%	95.17%

**Table 11:** Statistical significance analysis comparing the standard equal-weight ensemble and the proposed TAWE.

Comparison	Statistical Test	*p*-Value	Interpretation
Age estimation (MAE)	Wilcoxon signed-rank	0.5816	Not statistically significant (*p* > 0.05)
Gender classification (Acc)	Exact McNemar	0.0166	Statistically significant (*p* < 0.05)

**Table 12:** Task-specific model weights used in the proposed TAWE.

Model	Age Estimation Weight	Gender Classification Weight
ViT	0.25	0.55
Swin Transformer	0.55	0.25
DeiT	0.20	0.20

**Table 13:** TAWE performance across age subgroups in the independent UTKFace test set.

Age Group	*N*	Age MAE	Age RMSE	Gender Acc (%)	Macro-F1 (%)
21–30	1537	3.9919	5.3426	94.99	94.82
31–40	865	4.5907	5.6707	95.14	95.04
41–50	452	6.0328	7.5344	94.25	93.04
51–60	431	7.4988	9.6405	94.43	93.36

**Table 14:** TAWE performance across ethnicity-code subgroups in the independent UTKFace test set. Note: Ethnicity is reported using the numeric codes embedded in the UTKFace filenames because the prediction files used in the present analysis do not contain textual ethnicity labels. The numeric codes are therefore retained to avoid introducing an unverified mapping between ethnicity codes and textual category names.

Ethnicity Code	*N*	Age MAE	Age RMSE	Gender Acc (%)	Macro-F1 (%)
0	1235	5.1853	6.8208	95.55	95.47
1	776	5.0560	6.7459	93.17	93.17
2	426	3.8335	5.0740	94.60	94.48
3	643	4.9160	6.4211	95.80	95.70
4	205	4.6045	5.9076	94.63	94.63

**Table 15:** TAWE performance by dataset gender label in the independent UTKFace test set.

Dataset Gender Label	*N*	Age MAE	Age RMSE	Correct Gender Classification (%)
Male (0)	1748	4.9442	6.3965	95.08
Female (1)	1537	4.8295	6.5472	94.60

**Table 16:** Computational complexity comparison of the evaluated transformer models and the proposed TAWE framework.

Model	Parameters (M)	FLOPs (G)	Model Size (MB)	GPU Memory (MB)	Inference Time (ms)
ViT-B/16	86.60	16.87	327.92	345.69	13.01
Swin-T	28.30	4.51	105.83	132.38	13.30
DeiT-B	86.53	17.02	327.58	344.92	13.18
Proposed TAWE	201.43	38.40	761.33	822.99	39.49

**Table 17:** Representative qualitative prediction examples obtained using the proposed TAWE on the UTKFace test set. For each representative sample, the input facial image, ground-truth (GT) age and gender, predicted (Pred.) age and gender, and the corresponding absolute age estimation error are presented. The selected examples include both successful predictions and representative failure cases.

Sample	Image	GT Age	Pred. Age	Abs Error	GT Gender	Pred. Gender
1-Success	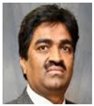	46	46	0	Male	Male
2-Success	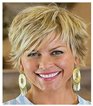	51	52	1	Female	Female
3-Success	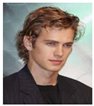	24	26	2	Male	Male
4-Failure	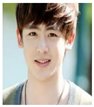	21	24	3	Male	Female
5-Failure	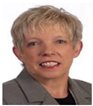	45	51	6	Female	Male
6-Failure	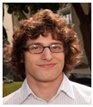	28	33	5	Male	Female

## Data Availability

The dataset used in this study is publicly available for non-commercial research purposes. The UTKFace: Large Scale Face Dataset, which supports the findings of this study, is available online at: https://susanqq.github.io/UTKFace/ (accessed on 30 June 2026).

## Abbreviations

The following abbreviations are used in this manuscript:

AI	Artificial Intelligence
CNN	Convolutional Neural Network
ViT	Vision Transformer
Swin Transformer	Shifted Window Transformer
DeiT	Data-efficient Image Transformer
TAWE	Task-aware Weighted Ensemble
MTL	Multi-Task Learning
MAE	Mean Absolute Error
RMSE	Root Mean Squared Error
MSE	Mean Squared Error
AdamW	Adaptive Moment Estimation with decoupled weight decay
UTKFace	UTKFace: Large Scale Face Dataset
XAI	Explainable Artificial Intelligence
